# Broad-spectrum suppression of bacterial pneumonia by aminoglycoside-propagated *Acinetobacter baumannii*

**DOI:** 10.1371/journal.ppat.1008374

**Published:** 2020-03-13

**Authors:** M. Indriati Hood-Pishchany, Ly Pham, Christiaan D. Wijers, William J. Burns, Kelli L. Boyd, Lauren D. Palmer, Eric P. Skaar, Michael J. Noto

**Affiliations:** 1 Department of Pediatrics, Division of Infectious Diseases, Boston Children’s Hospital, Boston, Massachusetts, United States of America; 2 Department of Pathology, Microbiology, and Immunology, Vanderbilt University Medical Center, Nashville, Tennessee, United States of America; 3 Vanderbilt Institute for Infection, Immunology, and Inflammation, Vanderbilt University Medical Center, Nashville, Tennessee, United States of America; 4 Tennessee Valley Healthcare System, US Department of Veterans Affairs, Nashville, Tennessee, United States of America; 5 Department of Medicine, Division of Allergy, Pulmonary, and Critical Care Medicine, Vanderbilt University Medical Center, Nashville, Tennessee, United States of America; Case Western Reserve University School of Medicine, UNITED STATES

## Abstract

Antimicrobial resistance is increasing in pathogenic bacteria. Yet, the effect of antibiotic exposure on resistant bacteria has been underexplored and may affect pathogenesis. Here we describe the discovery that propagation of the human pathogen *Acinetobacter baumannii* in an aminoglycoside antibiotic results in alterations to the bacterium that interact with lung innate immunity resulting in enhanced bacterial clearance. Co-inoculation of mice with *A*. *baumannii* grown in the presence and absence of the aminoglycoside, kanamycin, induces enhanced clearance of a non-kanamycin-propagated strain. This finding can be replicated when kanamycin-propagated *A*. *baumannii* is killed prior to co-inoculation of mice, indicating the enhanced bacterial clearance results from interactions with innate host defenses in the lung. Infection with kanamycin-propagated *A*. *baumannii* alters the kinetics of phagocyte recruitment to the lung and reduces pro- and anti-inflammatory cytokine and chemokine production in the lung and blood. This culminates in reduced histopathologic evidence of lung injury during infection despite enhanced bacterial clearance. Further, the antibacterial response induced by killed aminoglycoside-propagated *A*. *baumannii* enhances the clearance of multiple clinically relevant Gram-negative pathogens from the lungs of infected mice. Together, these findings exemplify cooperation between antibiotics and the host immune system that affords protection against multiple antibiotic-resistant bacterial pathogens. Further, these findings highlight the potential for the development of a broad-spectrum therapeutic that exploits a similar mechanism to that described here and acts as an innate immunity modulator.

## Author summary

Preserving the ability to treat infectious diseases with antibiotics in the face of the rapid proliferation of drug-resistant bacterial pathogens is among the greatest challenges facing medicine. Efforts to combat antimicrobial resistance may include strategies to maximize the utility of existing antibiotics while also identifying new therapeutic targets to treat bacterial infections. *Acinetobacter baumannii* is a human pathogen and strains of *A*. *baumannii* have acquired multi- and pan-antibiotic resistance. Here, we demonstrate that *A*. *baumannii* that is resistant to the aminoglycoside class of antibiotics is rapidly cleared from the lungs of mice when exposed to aminoglycoside antibiotics. Exposure to aminoglycosides induces changes in *A*. *baumannii* that interact with mouse antibacterial defenses, leading to rapid clearance of the infection. Further, killed aminoglycoside-exposed *A*. *baumannii* interacts with innate immunity in the lung to enhance the clearance of other pathogenic bacteria. These findings indicate that pneumonia caused by aminoglycoside-resistant *A*. *baumannii* may be effectively treated with aminoglycoside antibiotics and also suggests that the host immune response can be targeted to enhance the clearance of bacterial infections.

## Introduction

Over the last century, antibiotics have revolutionized the treatment of infectious diseases; however, increasing rates of microbial resistance now limit the efficacy of the existing antibiotic armamentarium. The Review on Antimicrobial Resistance estimates that at least 700,000 deaths worldwide are attributable to infections caused by antimicrobial resistant pathogens annually and projects this number to increase more than ten-fold by 2050 [1]. *A*. *baumannii* is a Gram-negative, opportunistic human pathogen that is among the leading causes of infections in critically-ill persons worldwide [2, 3]. Pneumonia is the most common manifestation of *A*. *baumannii* disease, particularly among persons receiving invasive mechanical ventilation, but this pathogen is capable of infecting the urinary tract, bloodstream, and is a significant cause of wound infections, including burns and battlefield wounds [3–7]. As an emerging pathogen, *A*. *baumannii* has evolved multi- and pan-antibiotic resistant phenotypes that have rapidly disseminated in the healthcare setting, prompting the Centers for Disease Control and Prevention to list *A*. *baumannii* among a group of antibiotic resistant pathogens posing a serious threat to human health [8–13]. As a result, factors contributing to the emergence and dissemination of antibiotic resistance have been the subject of extensive study, while mechanisms underlying *A*. *baumannii* pathogenesis are less well understood.

Exposure of an antibiotic-resistant bacterium to an agent to which it is resistant may alter the physiology of the organism without eliminating the ability of the organism to replicate. Antibiotics retard the growth of resistant organisms, presumably due to fitness costs associated with maintenance and expression of resistance determinants [14–16]. Antibiotics may also directly affect pathogenesis by altering the interaction of the organism with the host. Growth of methicillin-resistant *S*. *aureus* in the presence of beta-lactam antibiotics results in reduced cross-linking of peptidoglycan in the cell wall. This altered peptidoglycan enhances MRSA pathogenicity by promoting inflammasome-induced proinflammatory signaling by the host [17]. In contrast, antibiotics can cooperate with innate immune effectors to enhance bacterial killing. Exposure to cell wall-active antibiotics enhances MRSA killing by the host-derived antimicrobial peptide, LL-37, and LL-37 killing of *Salmonella enterica* is enhanced upon exposure to multiple classes of antibiotics [18, 19]. Further, *A*. *baumannii* has been found to alter pilus expression, biofilm formation, and capsule production in response to various antibiotics [20, 21]. These findings demonstrate that antibiotics can have complex effects on host-pathogen interactions that extend beyond simply inhibiting bacterial growth.

Many prevalent antibiotic-resistant bacteria are opportunistic pathogens that are capable of causing disease only in a subset of persons with compromised host defenses. Therefore, bolstering innate immunity may be sufficient to eradicate these pathogens, and innate immunity-enhancing therapeutics may provide an antibiotic-independent treatment approach [22, 23]. Here we describe the discovery that propagation of *A*. *baumannii* in an aminoglycoside antibiotic results in alterations to the bacterium that interact with innate immunity in the lung in a manner that enhances clearance of multiple Gram-negative bacterial pathogens.

## Results

### *A*. *baumannii* Tn5A7 induces host-mediated enhanced clearance of WT *A*. *baumannii* in a murine pneumonia model

**Fig 1:**
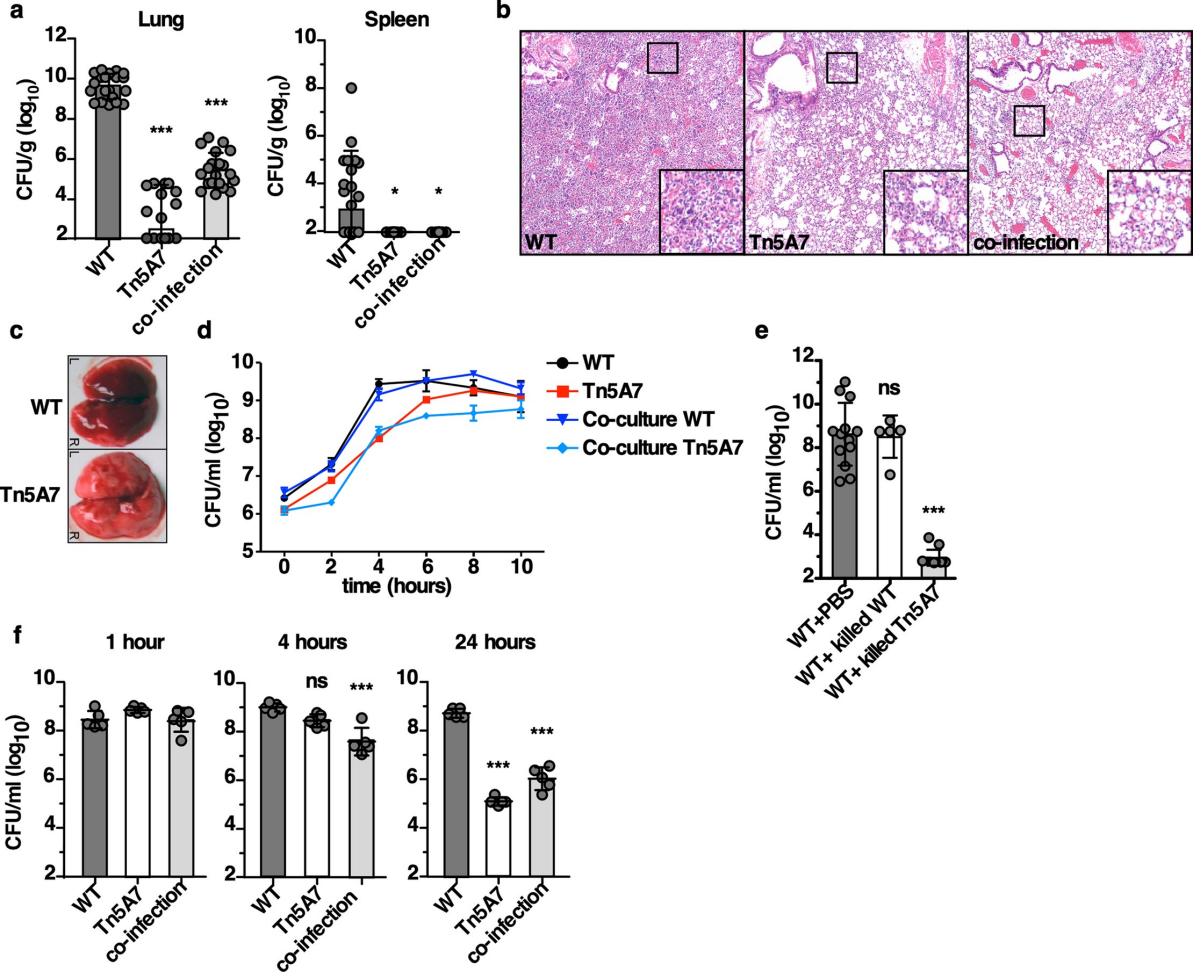
*A*. *baumannii* Tn5A7 enhances clearance of WT *A*. *baumannii* in a murine pneumonia model. (a) Mice were challenged intranasally with WT *A*. *baumannii*, Tn5A7, or an equal inoculum of both (co-infection) and bacterial burdens were enumerated from the lung and spleen at 36 hpi. (b) Representative hematoxylin and eosin-stained lung sections and (c) gross lungs are depicted from mice infected with WT *A*. *baumannii*, Tn5A7, or co-infected (H&E only) at 36 hpi. (d) WT and Tn5A7 were grown in lysogeny broth separately or in co-culture and bacteria were enumerated at the indicated time points and growth curves are shown. Points depict the mean and error bars represent standard deviation of three independent replicates per group. (e) WT and Tn5A7 were chemically killed and mixed with an equal inoculum of live WT prior to intranasal challenge and bacterial burdens in the lung at 36 hpi are shown. (f) Mice were infected with WT, Tn5A7, or co-infected and bacterial burdens in the lungs are shown at 1, 4, and 24 hpi are shown. For all experiments, WT was propagated in LB medium while Tn5A7 was propagated in LB containing kanamycin to maintain selective pressure prior to infection. Circles represent individual animals, columns depict the mean, and error bars show standard deviation of the mean. (a, e, and f) Means were compared with the mean of the first column using a one-way ANOVA adjusted for multiple comparisons. CFU/ml, colony forming units per milliliter; CFU/g, colony forming units per gram of organ homogenate; *, P<0.05; **, P<0.01; ***, P<0.001.

Given the known role for lipopolysaccharide (LPS) in inducing inflammation, a Tn5 transposon mutant with a disruption in *lpsB* was selected to investigate the role of LPS in *A*. *baumannii* pathogenesis using a murine pneumonia model [24]. *lpsB* encodes a glycosyltransferase involved in the biosynthesis of the core component of LPS [25]. Consistent with the previously described role for *lpsB* in pathogenesis, the transposon mutant, Tn5A7, exhibited a profound defect in virulence with a 7-log_10_ reduction in bacterial burdens in the lung at thirty-six hours post-infection relative to the wild-type strain Ab 17978 (Fig 1A). Notably, Tn5A7 produced a marked reduction in neutrophilic and necrotizing bronchopneumonia with interstitial consolidation (Fig 1B) that is evident on gross examination of the lungs (Fig 1C). Because LPS is a potent proinflammatory stimulus and some degree of inflammation may benefit a pathogen during infection [26, 27], an equal inoculum of WT and Tn5A7 were used for co-infection to determine if the presence of intact LPS from the WT strain complemented the virulence defect of Tn5A7. Surprisingly, Tn5A7 markedly attenuated WT infection with over a 4-log_10_ reduction in WT bacterial burdens in the lung at 36 hours (Fig 1A), and a reduction in neutrophilic and necrotizing bronchopneumonia that mirrors the findings for Tn5A7 mono-infection (Fig 1B). These data demonstrate that the presence of Tn5A7 leads to reductions in WT bacterial burdens and histopathologic findings of pneumonia during co-infection of the murine lung.

One possible explanation for the reduction in WT bacterial burdens during co-infection with Tn5A7 is direct antagonism of WT growth by Tn5A7. Interbacterial antagonism was not seen when these strains were co-cultured in rich medium as the presence of Tn5A7 did not alter WT growth (Fig 1D). To ensure that active inter-bacterial interactions were not responsible for the reduced WT bacterial burdens *in vivo*, each strain was chemically killed prior to intranasal co-infection. Chemically killed Tn5A7, but not chemically killed WT, enhanced clearance of WT infection in the lung (Fig 1E). These findings indicate that the enhanced clearance of WT infection by Tn5A7 does not result from active inter-bacterial interactions and is dependent upon the host response. It is possible that Tn5A7 alters WT colonization or otherwise changes infection kinetics. To test this hypothesis, mice were infected with WT or Tn5A7 alone, or co-infected with both strains and bacterial burdens in the lung were monitored at one, four, and twenty-four hours post-infection. Tn5A7 does not alter initial colonization as mice that were infected with WT, Tn5A7, or co-infected, had similar numbers of bacteria recovered from the lungs at one-hour post-infection. However, in the presence of Tn5A7, clearance of WT infection occurs as early as four hours post-infection (Fig 1F), suggesting innate immune mechanisms in the lung are responsible for this enhanced clearance. In contrast to the lung, Tn5A7 does not protect mice during systemic infection (S1 Fig). These data indicate that Tn5A7 interacts with host defenses to induce rapid clearance of WT *A*. *baumannii* from the lung.

### Tn5A7-mediated clearance of WT from the lung is dependent upon aminoglycoside exposure and independent of the gene disrupted by transposon insertion

**Fig 2:**
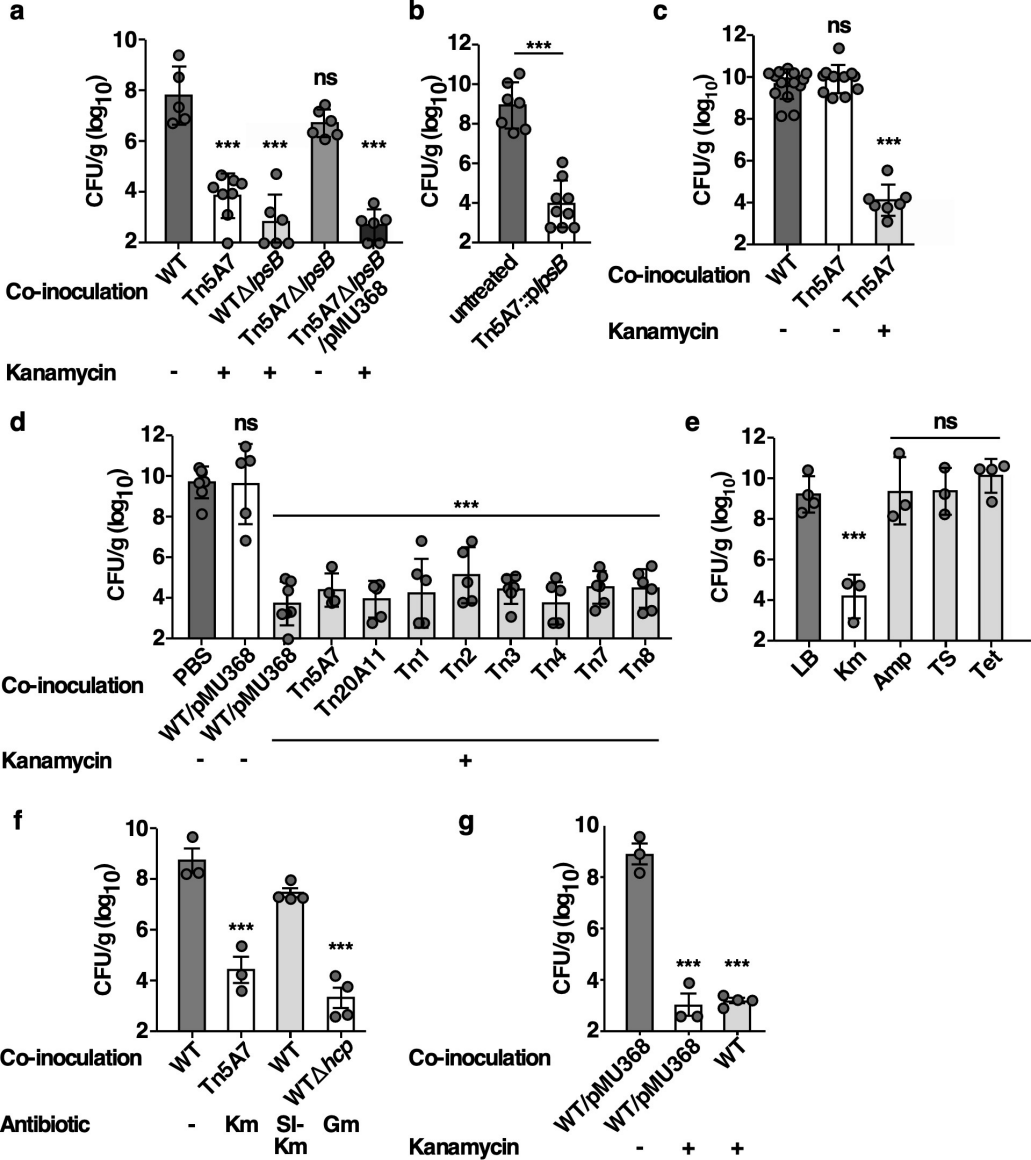
Propagation of *A*. *baumannii* in kanamycin prior to infection induces antibacterial innate immune interactions. (a) Mice were infected with WT mixed with the chemically killed strains indicated. Propagation in the presence or absence of kanamycin in the growth medium prior to infection is depicted below the graph. (a) Mice were challenged intranasally with WT *A*. *baumannii* propagated in lysogeny broth prior to infection and mixed with PBS or with chemically killed Tn5A7 carrying *lpsB* on a plasmid that had been propagated in lysogeny broth containing kanamycin prior to killing. (c) Mice were challenged with WT mixed with an equal volume of chemically killed Tn5A7 propagated with and without kanamycin prior to infection. (d) Mice were infected with WT mixed with an equal volume of PBS or the chemically killed strains listed. Propagation in the presence or absence of kanamycin in the growth medium prior to infection is depicted below the graph. (e) Mice were infected with WT propagated in lysogeny broth prior to infection that was mixed with a chemically killed derivative of Tn5A7 that was propagated in lysogeny broth alone or containing kanamycin (Km), ampicillin (Amp), trimethoprim and sulfamethoxazole (TS), or tetracycline (Tet) prior to infection. (f) Mice were infected with WT propagated in lysogeny broth prior to infection that was mixed with chemically killed WT grown in lysogeny broth, chemically killed Tn5A7 grown in kanamycin, chemically killed WT grown in a sub inhibitory concentration of kanamycin (SI-Km, 0.625 μg/mL), or WTΔ*hcp* grown in gentamicin. (g) Mice were infected with WT propagated in lysogeny broth that was mixed with chemically killed WT/pMU368 propagated in the presence or absence of kanamycin or with killed WT that was propagated in lysogeny broth prior treatment with 40 μg/mL of kanamycin for 3 hours. For panels a—g mice were challenged intranasally and bacterial burdens in the lung at 36 hpi are depicted. Circles represent individual animals, columns depict the mean, and error bars show standard deviation of the mean. Means were compared with the mean of the first column using a one-way ANOVA adjusted for multiple comparisons. CFU/g, colony forming units per gram of organ homogenate *, P<0.05; **, P<0.01; ***, P<0.001; ns, not statistically significant.

Tn5A7-mediated clearance of WT from the lung may be a consequence of *lpsB* disruption or from antibiotic selection used prior to Tn5A7 outgrowth on the day of infection. To distinguish these hypotheses, allelic exchange was used to replace *lpsB* with a kanamycin-resistance cassette in WT [24] and *lpsB* was complemented in Tn5A7 by expression of *lpsB in trans*. Similar to Tn5A7, WTΔ*lpsB* enhanced the clearance of WT infection during co-inoculation (Fig 2A). However, complementation of *lpsB* did not reverse the ability of Tn5A7 to enhance clearance of WT infection (Fig 2B), suggesting that disruption of *lpsB* in Tn5A7 is not responsible for the enhanced clearance of WT infection. To further address the role of *lpsB* in the enhanced clearance of WT *A*. *baumannii*, *lpsB*, and the Tn5 cassette contained within it, were deleted and replaced by *tetA* in Tn5A7 (Tn5A7Δ*lpsB*). Mice infected with WT mixed with killed Tn5A7Δ*lpsB* did not exhibit enhanced clearance of WT infection (Fig 2A), indicating that the Tn5 insertion is required for Tn5A7-mediated enhanced clearance of WT *A*. *baumannii*. As the Tn5 insertion encodes a kanamycin-resistance determinant and kanamycin was used as selection for resistant strains prior to the outgrowth on the day of infection, pMU368 was introduced into Tn5A7Δ*lpsB* to provide plasmid-borne kanamycin resistance. Killed, kanamycin-propagated Tn5A7Δ*lpsB*/pMU368 enhanced the clearance of WT during co-infection, suggesting that exposure to kanamycin is responsible for the Tn5-induced enhanced bacterial clearance from the lung. Next, Tn5A7 propagated in the presence, but not the absence, of kanamycin, enhanced the clearance of the WT during co-infection (Fig 2C). Further, seven additional Tn5 mutants with intact *lpsB* and WT/pMU368 grown in kanamycin enhance the clearance of WT infection (Fig 2D). These findings indicate that exposure to kanamycin is responsible for the enhanced clearance of WT infection induced by Tn5A7 and WTΔ*lpsB*.

Exposure to kanamycin induces changes to *A*. *baumannii* that interact with the host in a manner that enhances bacterial clearance from the lung. To determine if these findings are unique to kanamycin or are a more conserved response to antibiotics, a derivative of Ab 17978 resistant to multiple antibiotics, Tn5A7Δ*pilQ*/pAT02, was grown in LB alone or LB supplemented with kanamycin, ampicillin, trimethoprim and sulfamethoxazole, or tetracycline prior to co-inoculation of mice with the WT strain. Growth in kanamycin, but not other antibiotics, resulted in enhanced clearance of WT infection, suggesting that kanamycin-induced changes to *A*. *baumannii* are not recapitulated by the other classes of antibiotics tested (Fig 2E). To determine if this enhanced bacterial clearance can be induced by growth in a different aminoglycoside antibiotic, a WT derivative resistant to gentamicin, WTΔ*hcp*, was grown in gentamicin and chemically killed prior to co-inoculation with WT grown in LB alone. Growth in gentamicin induced enhanced WT clearance from the lung, suggesting that growth with aminoglycoside antibiotics alter *A*. *baumannii* in a manner that interacts with the antibacterial immune response in the lung (Fig 2F). To determine if resistance to kanamycin is required to induce enhanced bacterial clearance from the lungs, WT was grown in a sub-inhibitory concentration of kanamycin, chemically killed, and co-inoculated with WT grown in LB alone. Growth in a sub-inhibitory concentration of kanamycin did not recapitulate the enhanced bacterial clearance induced by Tn5A7 grown in a higher concentration of kanamycin (Fig 2F, S2 Fig). This finding may indicate that either higher concentrations of kanamycin in the growth medium or a kanamycin resistance determinant is required to induce enhanced bacterial clearance from the lung. To distinguish these possibilities, WT was grown to mid-logarithmic phase in LB alone at which point kanamycin was added to the medium. Following incubation with kanamycin, the bacteria were killed and used to co-inoculate mice with live WT propagated in LB. Co-inoculation with killed WT that had been incubated in kanamycin resulted in enhanced clearance of the live strain, indicating that kanamycin-resistance is not required for the enhanced clearance of the WT strain and exposure to kanamycin is sufficient (Fig 2G). In total, these data indicate that exposure of *A*. *baumannii* to an aminoglycoside antibiotic induces changes that interact with innate immunity to enhance the clearance of a co-infecting strain.

### Aminoglycoside exposure results in enhanced clearance of aminoglycoside-resistant *A*. *baumannii*

**Fig 3:**
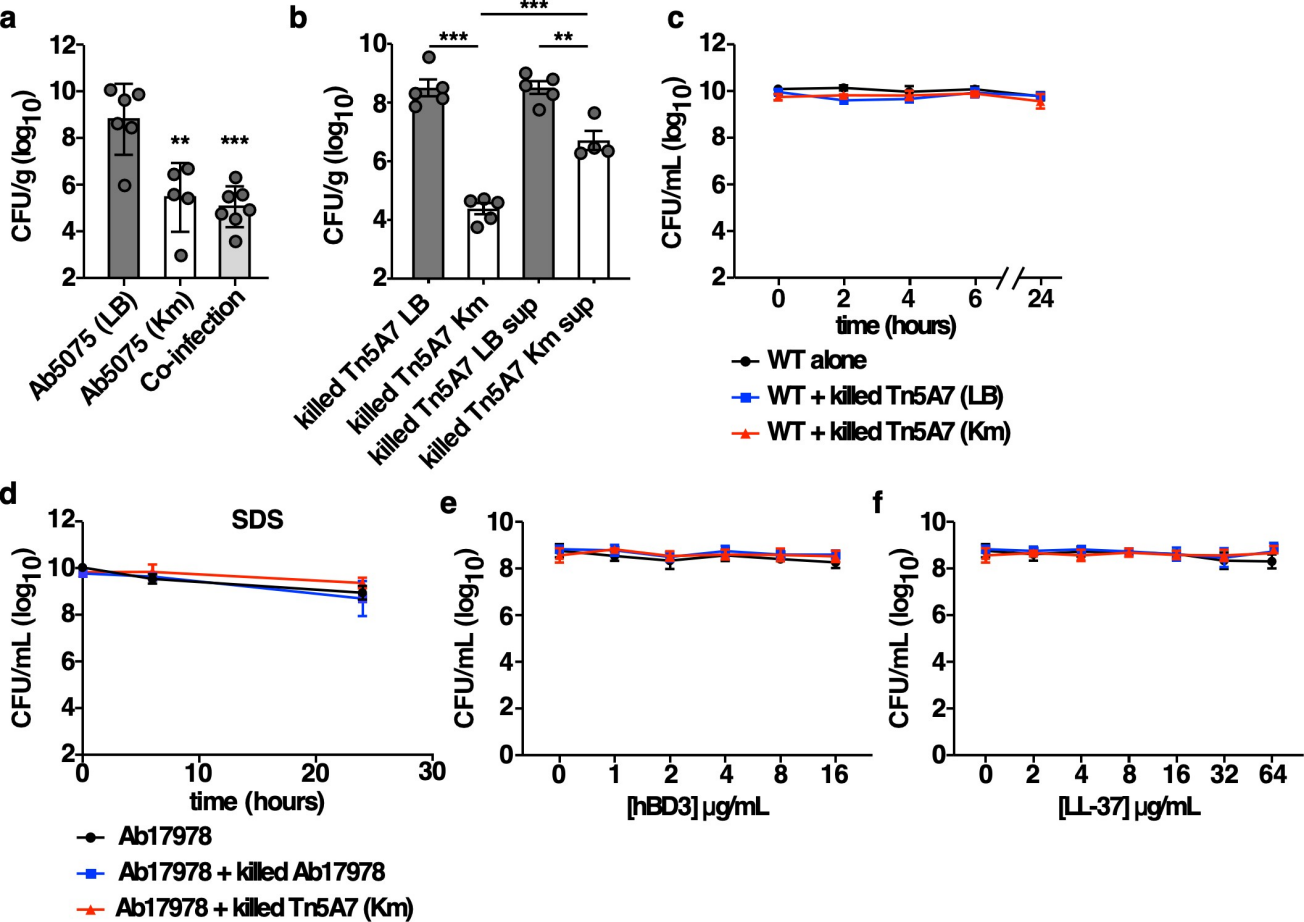
Aminoglycoside exposure results in enhanced clearance of aminoglycoside-resistant *A*. *baumannii*. (a) Mice were infected with Ab5075 propagated in lysogeny broth with and without kanamycin or co-infected with Ab5075 propagated in lysogeny broth mixed with killed Ab5075 propagated in lysogeny broth containing kanamycin. (b) Mice were infected with WT propagated in lysogeny broth mixed with killed Tn5A7 propagated in the presence and absence of kanamycin or the filter-sterilized supernatant of the Tn5A7 cell suspension. For panels a and b mice were challenged intranasally and bacterial burdens in the lung at 36 hpi are depicted. (c) The indicated bacterial strains were prepared in the same manner as used to infect mice and the viability of the live WT strain in the PBS suspension was assessed over time. (d) Bacteria were prepared as in (c) except that 0.01% sodium dodecyl sulfate was added to the suspension and the viability of WT was monitored over time. (d and e) Bacteria were prepared as in (c) and the indicated concentrations of human β-defensin 3 (hBD3) or LL-37 were added to the suspension and the viability of the WT strain was assessed after 90 minutes of incubation. Circles represent individual animals, columns depict the mean, and error bars show standard deviation of the mean. Means were compared with the mean of the first column unless otherwise indicated using a one-way ANOVA adjusted for multiple comparisons. CFU/g, colony forming units per gram of organ homogenate; CFU/mL, colony forming units per mL of medium; **, P<0.01; ***, P<0.001.

To determine if co-inoculation with a kanamycin-propagated strain enhances the clearance of a kanamycin-resistant strain from the lungs of mice, an *A*. *baumannii* strain with enhanced virulence in a murine pneumonia model, Ab5075, was selected [28]. Kanamycin-propagated Ab5075 had a greater than 3-log_10_ reduction in bacterial burdens during mono-infection and co-inoculation with killed, kanamycin-propagated Ab5075 resulted in a greater than 3-log_10_ reduction in Ab5075 propagated in medium alone (Fig 3A). To determine if the molecules responsible for the enhanced bacterial clearance induced by aminoglycoside-propagated *A*. *baumannii* are released into the supernatant, Tn5A7 was propagated with or without kanamycin, killed, and prepared for infection as described above. Either the killed bacterial suspension or the supernatant was mixed with the live strain prior to co-inoculation of mice. The kanamycin-propagated Tn5A7 supernatant resulted in a significant reduction in bacterial burdens relative to the non-kanamycin supernatant but the magnitude of this effect was significantly less than that achieved by the kanamycin-propagated Tn5A7 cell suspension (Fig 3B), indicating that the molecules responsible for the enhanced bacterial clearance are present in the supernatant. Aminoglycoside antibiotics are known to exhibit a post-antibiotic effect [29, 30]. It is possible that, despite washing, kanamycin is liberated from the kanamycin-propagated strain and the released kanamycin kills the kanamycin-susceptible WT strain when the strains are mixed prior to co-inoculation of mice. To test this hypothesis, Tn5A7 was propagated in the presence or absence of kanamycin, killed, washed, and mixed with live WT and the viability of the WT strain was followed over time. Co-confinement with kanamycin-propagated Tn5A7 did not result in enhanced killing of the WT strain (Fig 3C). Taken together, these data indicate that enhanced bacterial clearance from the lung is independent of kanamycin-mediated killing.

Aminoglycoside antibiotics are known to bind to the Gram-negative outer membrane and enhance membrane permeability [31, 32]. Therefore, sub-inhibitory concentrations of kanamycin liberated from killed, kanamycin-propagated *A*. *baumannii* may render the live strain susceptible to killing by innate immune effectors through the aminoglycoside-outer membrane interaction. To test this, WT was mixed with killed WT or killed kanamycin-propagated Tn5A7, exposed to the detergent sodium dodecyl sulfate (SDS, Fig 3D), or the antimicrobial peptides human β-defensin 3 (hBD3, Fig 3E) and LL-37 (Fig 3F), and bacterial viability was monitored. Exposure of the live WT strain to the killed kanamycin-propagated strain did not render the live strain susceptible to killing by SDS, hBD3, or LL-37. While these data do not support a model whereby liberated aminoglycoside renders the live strain susceptible to host-mediated killing by antimicrobial peptides, this possibility cannot be excluded.

### The enhanced clearance of WT infection is MyD88-dependent but independent of TLR4, TLR9, or IL-1 receptor signaling

**Fig 4:**
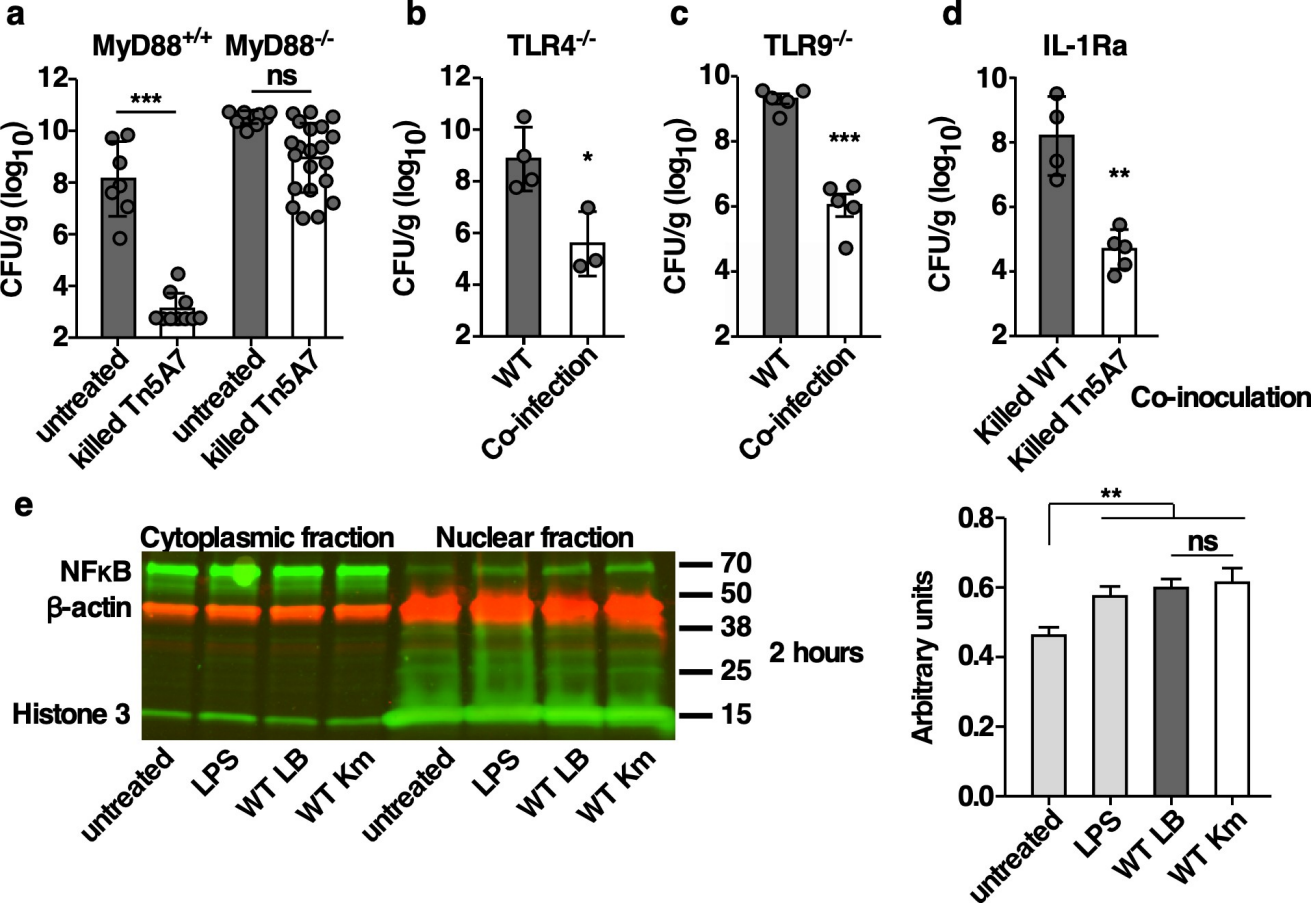
Kanamycin propagated *A*. *baumannii*-mediated enhanced clearance of WT infection is MyD88-dependent but independent of TLR4, TLR9, or IL-1 signaling. (a) MyD88^+/+^ or MyD88^-/-^ mice were infected with WT propagated in lysogeny broth mixed with PBS or mixed with chemically killed Tn5A7 propagated in lysogeny broth containing kanamycin prior to infection. (b) TLR4^+/+^ or TLR4^-/-^ mice were infected with WT propagated in lysogeny broth mixed with PBS or mixed with chemically killed Tn5A7 propagated in lysogeny broth containing kanamycin prior to infection. (c) TLR9^+/+^ or TLR9^-/-^ mice were infected with WT propagated in lysogeny broth mixed with PBS or mixed with chemically killed Tn5A7 propagated in lysogeny broth containing kanamycin prior to infection. (d) Mice were treated with 200 μg of the IL-1 receptor antagonist, anakinra, two days prior and on the day of infection with WT propagated in lysogeny broth mixed with PBS or mixed with chemically killed Tn5A7 propagated in lysogeny broth containing kanamycin prior to infection. (e) RAW264.7 cells were treated with LPS or infected with *A*. *baumannii* grown in the presence or absence of kanamycin prior to infection and nuclear localization of NFκB was assessed by Western blotting at two hours post-infection. A representative gel image and densitometric quantification of three independent replicates are shown. For panels a, b, c, and d, mice were challenged intranasally and bacterial burdens in the lung at 36 hpi are depicted. Circles represent individual animals, columns depict the mean, and error bars show standard deviation of the mean. Means were compared with the mean of the first column using a one-way ANOVA adjusted for multiple comparisons (e) or Welch’s t-test (a, b, c, and d). CFU/g, colony forming units per gram of organ homogenate; β-actin is a loading control; Histone 3 is a control for nuclear localization; *, P<0.05; **, P<0.01; ***, P<0.001; ns, not statistically significant.

Co-inoculation of mice with aminoglycoside-propagated *A*. *baumannii* induces host-mediated enhanced bacterial clearance that may result from interactions between an aminoglycoside induced pathogen-associated molecular pattern (PAMP) and host pattern recognition receptors (PRRs). MyD88 is an adaptor protein that is required for signaling downstream of PRRs, including many TLRs and the IL-1 receptor family [33–35]. MyD88^+/+^ and MyD88^-/-^ mice were infected with WT alone or co-inoculated with WT and killed Tn5A7 that had been grown in kanamycin prior to infection. MyD88^+/+^ but not MyD88^-/-^ mice exhibited enhanced clearance of the WT strain from the lung when co-inoculated with killed Tn5A7 grown in kanamycin (Fig 4A), indicating that MyD88 signaling is required for the enhanced bacterial clearance induced by aminoglycoside-propagated *A*. *baumannii*. TLR4, TLR9, and inflammasome signaling have been shown to alter the course of *A*. *baumannii* pneumonia [36–38]. However, co-inoculation with Tn5A7 resulted in enhanced clearance of WT infection in both *TLR4*^*-/-*^ and *TLR9*^*-/-*^ mice (Fig 4B and 4C), indicating that neither TLR4 nor TLR9 signaling is required for differential recognition of kanamycin-propagated *A*. *baumannii* by the host. To determine if IL-1 signaling is required for differential recognition of kanamycin-propagated *A*. *baumannii* by the host, mice were treated with the IL-1R antagonist, anakinra, prior to infection with WT alone or co-inoculation with WT and killed Tn5A7 that had been grown in kanamycin prior to infection. Treatment with anakinra did not alter Tn5A7-mediated enhanced clearance of WT infection (Fig 4D). Therefore IL-1R signaling is not required for differential recognition of kanamycin-propagated *A*. *baumannii* by the host. Signaling downstream of MyD88 results in the activation and nuclear localization of pro-inflammatory transcription factors, such as NFκB. Given the demonstrated role for MyD88 signaling in vivo, differential activation of NFκB by kanamycin-propagated *A*. *baumannii* was assessed in vitro. Macrophage-like RAW264.7 cells were infected with *A*. *baumannii* grown in the presence or absence of kanamycin and nuclear localization of NFκB was assessed by Western blotting. Surprisingly, RAW264.7 cells infected with kanamycin-propagated *A*. *baumannii* do not exhibit increased NFκB activation relative to cells infected with LB-propagated strains at 2 hours (Fig 4E), or at 30 minutes, 1 hour, or 4 hours following infection. This finding suggests that aminoglycoside-propagated *A*. *baumannii* does not induce differential NFκB activation in macrophages. Therefore, MyD88 and NFκB signaling may be required for host defense against *A*. *baumannii*, but the enhanced bacterial clearance induced by aminoglycoside-propagated *A*. *baumannii* does not involve differential signaling through these pathways.

### Aminoglycoside-propagated *A*. *baumannii* alters cytokine and chemokine production

**Fig 5:**
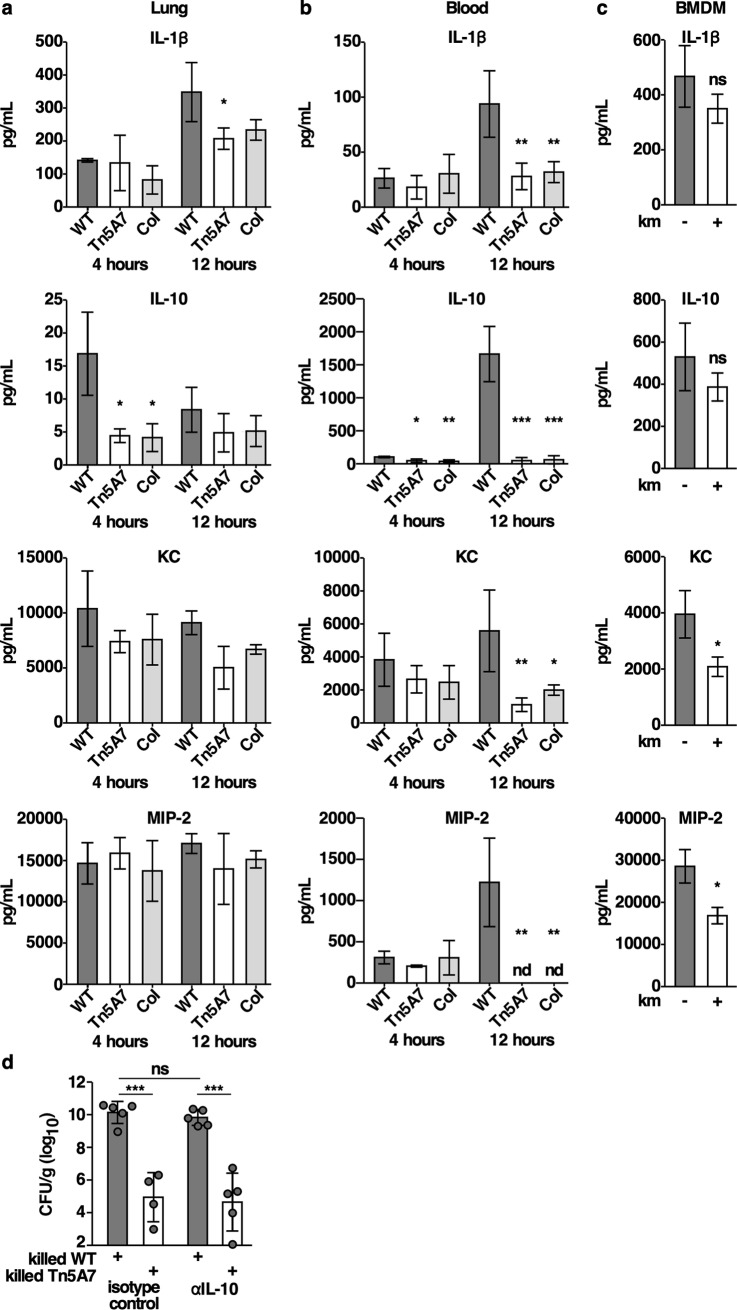
Infection with kanamycin propagated *A*. *baumannii* results in reduced cytokine and chemokine production in the lung and blood. The indicated cytokines and chemokines were quantified from whole lung homogenates (a) and blood (b) of mice at four- and 12-hours following infection with WT propagated in lysogeny broth, Tn5A7 propagated in lysogeny broth containing kanamycin, or co-infected with an equal mixture of the two strains. (c) BMDMs were infected with the indicated bacterial strains at a multiplicity of infection of 15. Infected cells were incubated for 4 hours at which time the cell culture supernatant was removed and assayed. (d) Mice were treated with intraperitoneal injection of 50 μg of anti-IL-10 antibody or isotype control at the time of infection with WT mixed with killed WT or mixed with killed Tn5A7 propagated in medium containing kanamycin. Columns depict the mean and error bars show standard deviation of the mean. Means were compared with the mean of WT at that time using a one-way ANOVA adjusted for multiple comparisons. *, P<0.05; **, P<0.01; ***, P<0.001; ns, not statistically significant.

Infection with kanamycin-propagated *A*. *baumannii* may alter cytokine and chemokine production and thereby augment the antibacterial immune response in the lung. A panel of cytokines and chemokines was quantified from lung homogenates and blood of mice infected with an equal inoculum of WT, Tn5A7 propagated in kanamycin, or co-infected with both strains. Bone marrow derived macrophages were also infected with WT/pMU368 grown in the presence or absence of kanamycin prior to infection. There was no significant difference in the levels of the proinflammatory cytokines, TNFα, IL-6, or INF-γ, induced by kanamycin-propagated strains relative to WT (S3 Fig). Mice infected with kanamycin-propagated *A*. *baumannii* exhibited reduced levels of IL-1β and IL-10 in the lungs and blood as well as reductions of the neutrophil chemokines KC and MIP-2 in the blood (Fig 5A and 5B). These findings were mirrored in vitro as BMDMs infected with kanamycin-propagated *A*. *baumannii* produced significantly less KC and MIP-2 (Fig 5C). It is possible that the reduction in the anti-inflammatory cytokine IL-10 produced in response to kanamycin propagated *A*. *baumannii* may contribute to the enhanced antibacterial immune response. Therefore, mice were treated with an IL-10 neutralizing antibody or isotype control at the time of infection with WT *A*. *baumannii* mixed with killed WT or killed Tn5A7 grown in the presence of kanamycin. Treatment with the IL-10 neutralizing antibody did not recapitulate the enhanced bacterial clearance induced by kanamycin propagated *A*. *baumannii* (Fig 5D), indicating that reduced IL-10 is not responsible for the enhanced bacterial clearance induced by exposure to *A*. *baumannii* grown in an aminoglycoside antibiotic.

### Aminoglycoside-propagated *A*. *baumannii* reduces early neutrophilic lung inflammation

**Fig 6:**
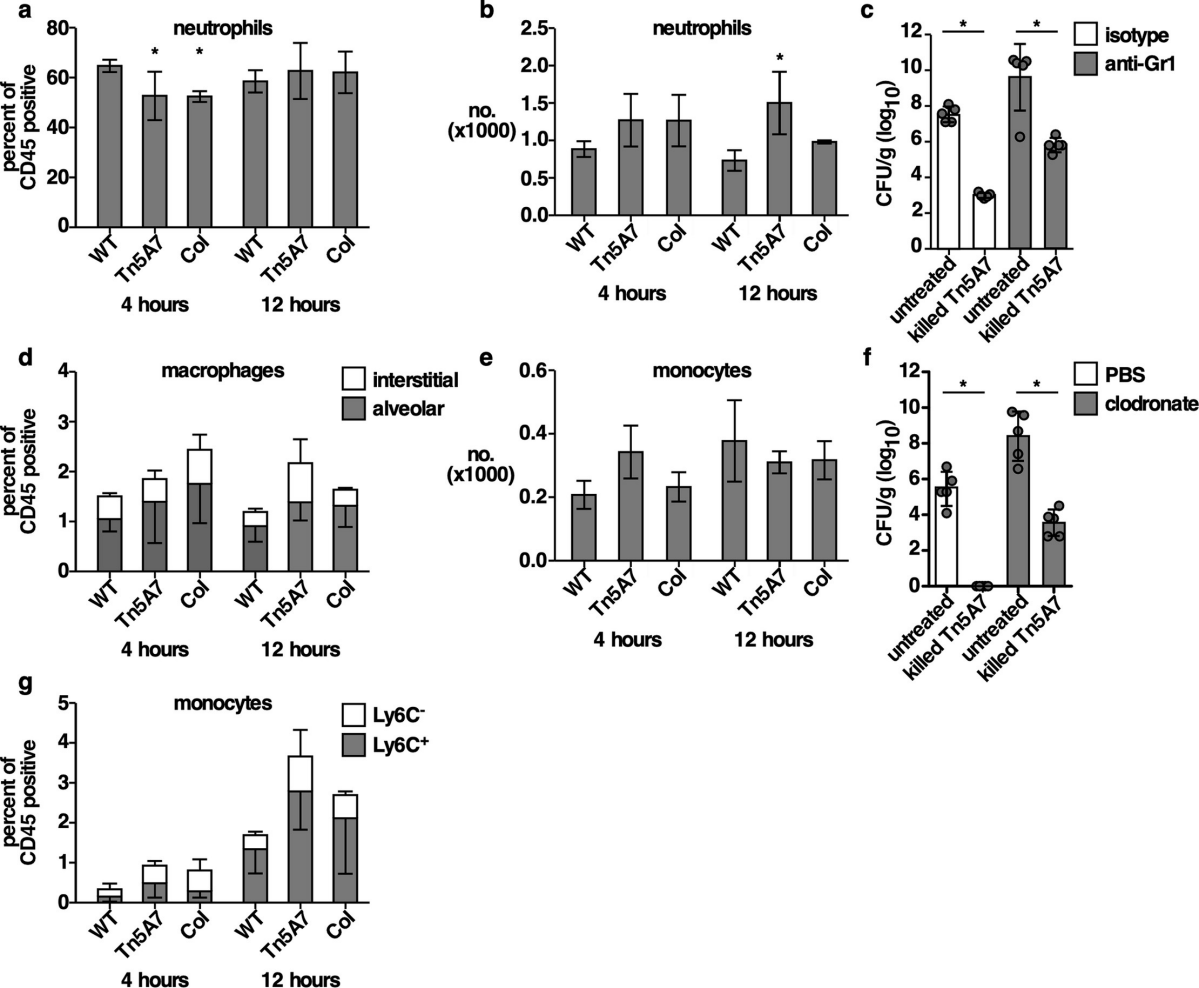
Kanamycin propagated *A*. *baumannii* alters neutrophil recruitment to the lung although neutrophils are dispensable for kanamycin propagated *A*. *baumannii*-mediated enhanced clearance of WT infection. (a, d, g) Neutrophils, macrophages, and monocytes were quantified from the lungs of mice using flow cytometry at four- and 12-hours following infection with WT *A*. *baumannii* propagated in lysogeny broth, Tn5A7 propagated in lysogeny broth containing kanamycin, or co-infected with an equal mixture of the two strains. The y-axis depicts the percentage all CD45-positive cells. (b and e) Neutrophils and monocytes were quantified from the blood of mice at four- and 12-hours following infection with WT propagated in lysogeny broth, Tn5A7 propagated in lysogeny broth containing kanamycin, or co-infected with an equal mixture of the two strains. (c) Neutrophils were depleted in mice through administration of an anti-Gr1 antibody or isotype control prior to infection with WT propagated in lysogeny broth and mixed with PBS or mixed with Tn5A7 propagated in lysogeny broth containing kanamycin. (f) Macrophages were depleted in mice through intranasal administration of clodronate liposomes or control liposomes prior to infection with WT propagated in lysogeny broth and mixed with PBS or mixed with Tn5A7 propagated in lysogeny broth containing kanamycin. For panels c and f, mice were challenged intranasally and bacterial burdens in the lung at 36 hpi are depicted. Circles represent individual animals, columns depict the mean, and error bars show standard deviation of the mean. Means were compared with the mean of WT at that time using a one-way ANOVA adjusted for multiple comparisons (a, b, d, e, and g) or Welch’s t-test (c and f). CFU/g, colony forming units per gram of organ homogenate; *, P<0.05; **, P<0.01; ***, P<0.001; ns, not statistically significant.

To determine if alterations to chemokine levels affected immune cell recruitment to the lungs of mice, immune cell infiltration to the lungs was assessed by flow cytometry using a myeloid antibody panel. Mice were infected with an equal inoculum of WT, Tn5A7 propagated in kanamycin, or co-infected with both strains, lungs were harvested for single cell analysis, and complete blood counts were performed at 4- and 12-hours post-infection. Representative contour plots of windows and gating strategy are shown in S4 Fig. Mice inoculated with kanamycin-propagated Tn5A7 or co-inoculated with Tn5A7 and WT exhibited a significant reduction in neutrophil infiltration to the lungs, but not blood, at 4 hours with a nonsignificant increase in macrophages and monocytes in the lungs but not blood (Fig 6A, 6B, 6D, 6E and 6G). Other immune cell populations in the lung that were discernable using this gating strategy, including eosinophils, natural killer cells, and lymphocytes, did not differ between conditions (S5 Fig). To determine if fluctuations in neutrophil or macrophage recruitment to the lung are responsible for the kanamycin-propagated *A*. *baumannii*-induced enhanced bacterial clearance, depletion strategies were employed. Neutrophils were depleted from mice by administration of an anti-Gr1 antibody and macrophages were depleted by intranasal instillation of clodronate liposomes prior to inoculation with WT or co-inoculation with WT and kanamycin-propagated Tn5A7. Although bacterial burdens in the lungs were higher when neutrophils or macrophages were depleted from mice, co-inoculation with Tn5A7 induced enhanced bacterial clearance in the lung despite depletion of neutrophils or macrophages (Fig 6C and 6F). These findings indicate that neutrophils and macrophages are dispensable for the enhanced bacterial clearance from the lungs induced by aminoglycoside-propagated *A*. *baumannii*.

### Aminoglycoside-propagated *A*. *baumannii* enhances the clearance of multiple Gram-negative bacterial pathogens during pneumonic infection

**Fig 7:**
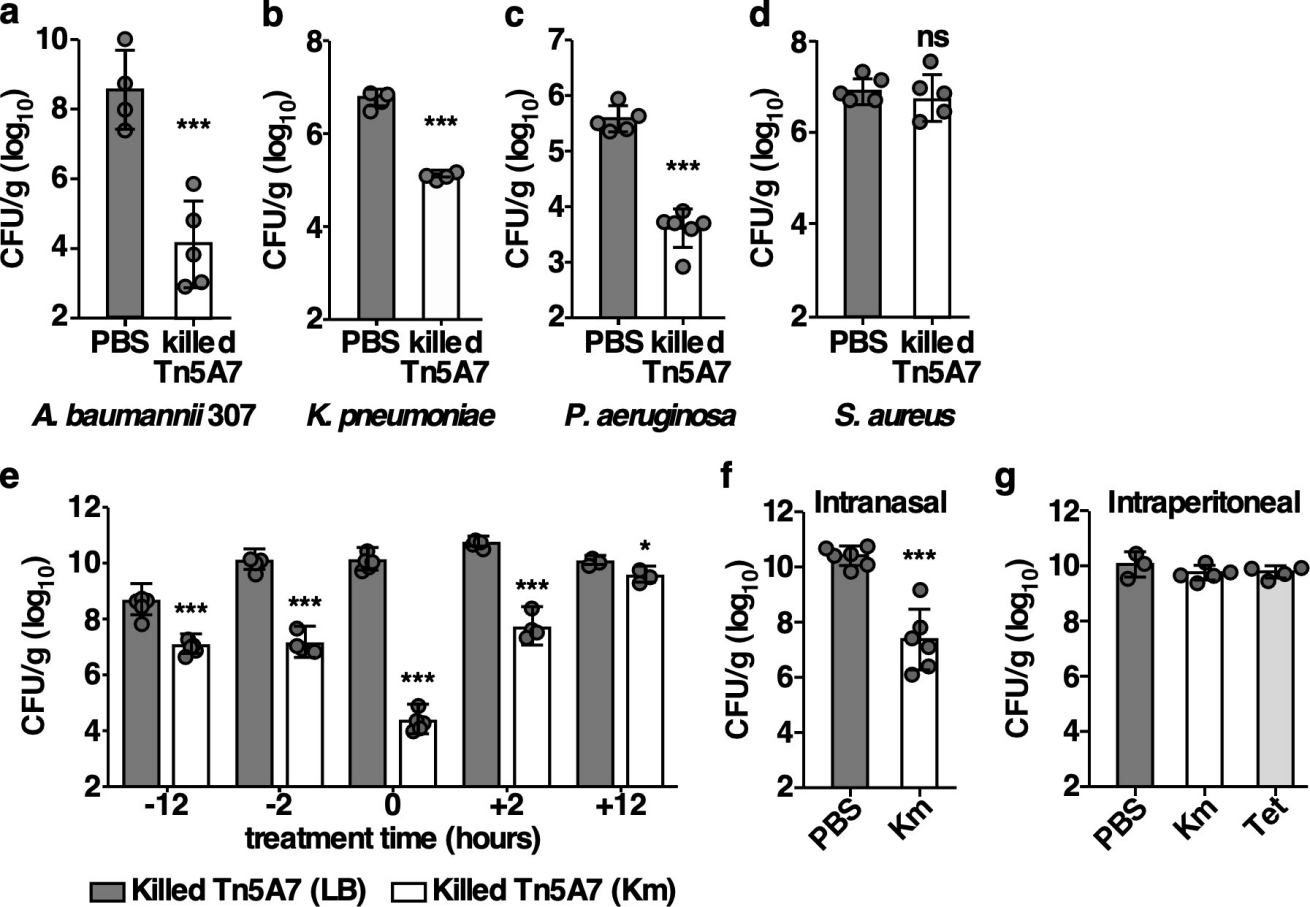
Kanamycin propagated *A*. *baumannii* enhance the clearance of multiple Gram-negative bacterial pathogens during pneumonic infection. (a) Mice were infected with *A*. *baumannii* 307 mixed with PBS or chemically killed Tn5A7 propagated in lysogeny broth containing kanamycin; (b) *K*. *pneumoniae* mixed with PBS or chemically killed Tn5A7 propagated in lysogeny broth containing kanamycin; (c) *P*. *aeruginosa* mixed with PBS or chemically killed Tn5A7 propagated in lysogeny broth containing kanamycin; or (d) *S*. *aureus* mixed with PBS or chemically killed Tn5A7 propagated in lysogeny broth containing kanamycin. (e) Mice were treated with intranasal administration of chemically killed Tn5A7 propagated in lysogeny broth with and without kanamycin at 12 or two hours prior to, at the time of, or two, or 12 hours after infection with WT *A*. *baumannii*. (f) Mice were infected with WT/pMU368 propagated in lysogeny broth followed by intranasal treatment with 40 mg/kg kanamycin or an equal volume of PBS. (g) Mice were infected with WT/p.*luxABCDE*.MU368.tet and treated with intraperitoneal injection of kanamycin (100 mg/kg), tetracycline (3 mg/kg), or an equal volume of PBS. (a-g) Mice were infected intranasally with the indicated pathogens and bacterial burdens were enumerated from the lung at 36 hpi (48 hpi for b). Means were compared using unpaired Welch’s *t*-test (a-d, and f) or one-way ANOVA adjusted for multiple comparisons (e and g). Unless otherwise noted, all comparisons were made with the first column of each graph. CFU/g, colony forming units per gram of organ homogenate; *, P<0.05; **, P<0.01; ***, P<0.001.

The dramatic effect of killed kanamycin-propagated *A*. *baumannii* on pneumonia caused by *A*. *baumannii* prompted investigations of other prominent lung pathogens. To assess the antibacterial spectrum of kanamycin-propagated *A*. *baumannii*, mice were infected with a different strain of *A*. *baumannii* (307) (Fig 7A), *K*. *pneumoniae* (Fig 7B), *P*. *aeruginosa* (Fig 7C), or *S*. *aureus* (Fig 7D) and either mock treated with PBS or treated with killed, kanamycin-propagated Tn5A7 at the time of infection. Treatment with killed Tn5A7 resulted in a nearly 4-log_10_ reduction in bacterial burdens in the lung for *A*. *baumannii* 307, a 2-log_10_ reduction in *P*. *aeruginosa* burdens in the lung, and a nearly 2-log_10_ reduction in *K*. *pneumonia* burdens. In contrast, treatment with killed Tn5A7 did not alter the course of *S*. *aureus* pneumonia. To further investigate the therapeutic potential of killed kanamycin-propagated *A*. *baumannii*, a treatment time course was performed. Treatment with killed, kanamycin propagated Tn5A7 at all time points enhanced clearance of WT *A*. *baumannii* (Fig 7E), although the magnitude of this effect was greatest when treatment occurred at the time of infection. Taken together, these findings demonstrate that chemically killed kanamycin-propagated *A*. *baumannii* afford significant protection against pneumonia caused by a wide-range of Gram-negative pathogens by enhancing bacterial clearance. Aminoglycoside antibiotics are used both topically, through inhaled administration, or systemically, through intravenous administration, for the treatment of bacterial pneumonia in humans [39]. To determine if aminoglycoside treatment of mice induces the enhanced clearance of aminoglycoside-resistant *A*. *baumannii*, mice were infected with a resistant strain of *A*. *baumannii* and treated with intranasal or intraperitoneal administration of kanamycin after infection. Intranasal, but not intraperitoneal, treatment of mice resulted in the enhanced clearance of aminoglycoside-resistant *A*. *baumannii* from the lungs (Fig 7F and 7G). The discrepancy between systemic and topical aminoglycoside treatment may reflect the higher local concentrations of kanamycin achieved by topical administration. Therefore, aminoglycoside treatment induces the clearance of aminoglycoside-resistant *A*. *baumannii* from the murine lung.

## Discussion

Infections caused by confirmed *Acinetobacter sp*. emerged in the 1960s and have increased in frequency in parallel with increasing complexity of patient care, including advances in, and increased use of, intensive care, and now are estimated to account for 1.8% of all healthcare-associated infections [3, 40–42]. *A*. *baumannii* is the most frequently isolated pathogen of this genus and multi-drug resistant isolates have been isolated from the environment and in association with agriculture and the food industry [42–45]. It is therefore possible that the rapid emergence of multi- and pan-drug resistant isolates of *A*. *baumannii* in the healthcare setting reflects selection of isolates existing in the environment. Here we report our finding that *A*. *baumannii* responds to the presence of aminoglycoside antibiotics by altering its physiology in a manner that interacts with antibacterial immunity in the murine lung. This finding adds to a growing body of literature indicating that *A*. *baumannii* may use the presence of antibiotics in the environment as a cue to alter its phenotype. Exposure of *A*. *baumannii* to sub-inhibitory concentrations of trimethoprim and sulfamethoxazole results in inhibition of Csu pilus expression and thereby reduces biofilm formation. Therefore, trimethoprim and sulfamethoxazole may serve as a signal for *A*. *baumannii* to disperse biofilms and thereby escape antibiotic pressure [20]. Many strains of *A*. *baumannii* constitutively express a type VI secretion system (T6SS), which is employed to kill competing bacteria within the environment [46]. T6SS expression is repressed by acquisition of a multi-drug resistance plasmid containing T6SS regulatory genes and, in the absence of selective antibiotic pressure, frequent plasmid loss occurs within the population. This plasmid loss results in T6SS expression by these antibiotic susceptible strains [47]. In this manner, the resistance plasmid provides a competitive advantage relative to other organisms when antibiotics are present in the environment and the absence of antibiotic pressure leads to loss of the resistance plasmid, T6SS expression, and a competitive advantage to antibiotic susceptible strains via T6SS-mediated interbacterial antagonism. Further, capsule biosynthesis in *A*. *baumannii* is directly regulated by exposure to antibiotics. Growth in sub-inhibitory concentrations of the protein synthesis inhibitors, chloramphenicol or erythromycin, increases capsular biosynthesis thereby increasing resistance to complement-mediated bacterial killing and enhancing virulence in a murine model of peritonitis [21]. It is clear from these data that *A*. *baumannii* has evolved not only the means to resist antibiotics but the capacity to detect antibiotics in the environment to regulate key biological functions.

In the clinical setting, resistance to antibiotics is defined based upon an organism’s ability to replicate in the presence of the antibiotic when cultured in laboratory conditions that do not reflect the conditions experienced during infection of a mammalian host. Herein, we have demonstrated that exposure of an aminoglycoside resistant isolate of *A*. *baumannii* to an aminoglycoside antibiotic results in a marked reduction in pathogenicity and essentially renders the strain avirulent in a murine model of pneumonia. This reduced pathogenesis is dependent upon interactions with the antibacterial host response in the lung. This finding bolsters the concept that antibiotics can sensitize resistant organisms to the host immune response, which has been demonstrated for host-derived antimicrobial peptides [18, 19]. Based upon these findings, it is likely that current laboratory-based testing underestimates the in vivo efficacy of existing antibiotics to combat multidrug-resistant pathogens. Further, the emergence of multi- and pan-antibiotic resistant bacterial pathogens has led to a critical need for novel therapeutic approaches to combat bacterial infections. Approaches that function to augment host defenses have been posited as potential strategies yet little progress has been made in this area [48–50]. Here we describe the discovery that aminoglycoside-propagated *A*. *baumannii* interacts with antibacterial immune response in the lung to result in the enhanced clearance of multiple clinically relevant Gram-negative pathogens. Previous work has demonstrated that stimulating inflammation in the lung with inhaled bacterial lysates or specific TLR agonists can enhance host defense against multiple pathogens [22, 51]. The potential limitation to this approach is that the increased non-specific inflammation may lead to collateral damage that impairs crucial lung functions, such as gas exchange. In contrast, aminoglycoside-propagated *A*. *baumannii* directly enhance antibacterial activity while simultaneously dampening the inflammatory response and resultant neutrophilic inflammation in the lung. This results in effective bacterial clearance, particularly during infection with WT *A*. *baumannii*, with less severe histologic evidence of lung injury, indicating that innate defenses in the lungs can be manipulated in nuanced ways to combat bacterial infection while limiting deleterious effects on the host.

The possibility that *A*. *baumannii* differentially interacts with the host immune response when propagated in an aminoglycoside antibiotic suggests that the aminoglycoside may induce the expression of a PAMP that is detected by the host. In this case, both the nature of this PAMP and the means by which it is recognized by the host are not known. TLR4, TLR9, and IL-1 are required for optimal host defense against *A*. *baumannii* [36–38, 52]; however, these receptor pathways are not required for the enhanced bacterial clearance induced by kanamycin-propagated *A*. *baumannii*. Aminoglycoside antibiotics are known to alter the release of pro-inflammatory molecules from Gram-negative bacteria. Aminoglycosides modulate the release of LPS [53], bind and neutralize LPS [54], and increase the release of membrane vesicles, which can alter the inflammatory response to infection [55]. The enhanced bacterial clearance induced by aminoglycoside-propagated *A*. *baumannii* was independent of LPS detection through TLR4 signaling (Fig 4B) and did not induced differential nuclear localization of the NFkB (Fig 4E), suggesting that aminoglycoside modulation of LPS release is not responsible for this phenotype. Although other pattern recognition receptors may be responsible for this phenotype, the alternative hypothesis that an aminoglycoside-propagated strain somehow sensitizes the co-infecting strain to aspects of innate immunity cannot be excluded. Aminoglycoside antibiotics are cationic and are known to bind to and permeabilize the outer membrane of Gram-negative organisms by displacing magnesium ions that allow for the tight packing of LPS in outer leaflet [31, 32]. It is possible that cell-associated aminoglycoside is liberated from the aminoglycoside-propagated bacterium and directly alters the physiology of the non-aminoglycoside propagated strain, thereby sensitizing it to innate immune effectors. However, exposure to a kanamycin-propagated strain did not increase the susceptibility of a non-kanamycin-propagated strain to killing by detergent or the antimicrobial peptides, LL-37 or human β-defensin 3, which target the cell envelope (Fig 3D, 3E and 3F). While the mechanisms remain to be determined, this work demonstrates an unrecognized cooperation between aminoglycoside antibiotics and host innate immunity to target a prominent multidrug-resistant bacterial pathogen. Furthermore, the finding that aminoglycoside-propagated *A*. *baumannii* alters host defense in the lung in a manner that enhances immune antibacterial activity while reducing inflammation supports the concept of host-mediated strategies to treat bacterial infection.

## Materials and methods

### Murine infection models

Wildtype, female, eight-week-old C57BL/6 mice were purchased from Jackson Laboratories. C3H/HeJ (TLR4^-/-^) mice were obtained from Jackson Laboratories. C3H control mice were obtained from Charles River Laboratories. TLR9^-/-^ mice in the C57BL/6 background were provided by Gregory B. Barton at the University of California, Berkeley [56, 57]. MyD88^-/-^ mice in the C57BL/6 background were provided by Sebastian Joyce at Vanderbilt University Medical Center [58]. The murine model of *A*. *baumannii* pneumonia was performed as previously described [59]. Briefly, *A*. *baumannii* strain ATCC 17978 was back-diluted 1:1000 from overnight culture and grown in lysogeny broth (LB) for 3.5 hours at 37°C with shaking, washed twice with cold PBS, and resuspended in PBS at an appropriate cell density for infection. Mice were infected intranasally with 3×10^8^ colony forming units *A*. *baumannii* in 30 μl PBS. At 36 hours post infection mice were euthanized and organs and blood were harvested. For co-infection experiments, individual strains were prepared as above, equal volumes of each strain were mixed (or AB17978 was mixed with an equal volume of PBS to ensure comparable numbers of AB17978 in each group), and 30 μl of the mixed inoculum was used to challenge mice intranasally. Following the 3.5-hour outgrowth, *A*. *baumannii* was chemically killed by adding an equal volume of ice-cold acetone: ethanol to the culture, incubating on ice for 5 minutes, pelleting cells, resuspending in ice-cold acetone: ethanol, pelleting cells, and washing twice with PBS prior to resuspending at the appropriate cell density for infection. *K*. *pneumoniae* pneumonia was performed as described above with an inoculum of 1x10^3^ CFU and the infection proceeded for 48 hours. *P*. *aeruginosa* pneumonia was performed as above with an inoculum of 1 to 5x10^7^ CFU. For *A*. *baumannii* systemic infection, Ab17978 and Tn5A7 was prepared in a similar manner and mice were infected retro-orbitally with 5×10^8^ colony forming units in 100 μl PBS as previously described [36]. Mice were treated with 200 μg of anakinra (SOBI) intraperitoneally 48 hours prior to and at the time of infection with Ab, with 40 mg/kg kanamycin in 30 μL of PBS intranasally 30 minutes post-infection, or with 100 mg/kg kanamycin or 3 mg/kg tetracycline in 200 μL of PBS intraperitoneally 30 minutes, 12, and 24 hours post-infection. Lungs, livers, and spleens were homogenized, serially diluted in PBS, and plated onto LBA for bacterial enumeration. For histological analyses, lungs were inflated with 1 ml of 10% formalin, fixed, embedded and stained as described previously [60]. Mice were randomized to treatment groups using the GraphPad QuickCalcs online randomization software available at (https://www.graphpad.com/quickcalcs/randomize1.cfm).

### Bacterial growth assays

Bacterial strains included in this study are listed in S1 Table. Bacteria were grown in lysogeny broth (LB) with shaking at 180 RPM or agar (LBA) at 37°C with antibiotic selection at the following concentrations as indicated: kanamycin 40 μg/mL, tetracycline 10 μg/mL, ampicillin 500 μg/mL, gentamicin 50 μg/mL, and 5 μg/mL of trimethoprim with 30 μg/mL of sulfamethoxazole. Bacterial strains were streaked onto LBA and incubated overnight at 37°C. A single colony was used to inoculate 3 ml of LB which was incubated for 20 hours at 37°C with shaking. Following incubation, the cultures were diluted 1:100 in 200 μl of LB in 96-well flat bottom tissue culture plates (Corning^™^ Costar^™^). The plate was incubated for 10 hours at 37°C with shaking and growth was assayed by determining the optical density at 600 nanometers at the indicated times using a BioTek Synergy 2 Multi-Mode Reader (BioTek) or by serial dilution in PBS and plating. Growth curves of all relevant strains in the presence or absence of kanamycin and gentamicin are depicted in S6 Fig. For in vitro survival experiments, *A*. *baumannii* strain ATCC 17978 and the indicated derivatives were back-diluted 1:1000 from overnight culture and grown in lysogeny broth (LB) with or without kanamycin 40 μg/mL for 3.5 hours at 37°C with shaking, washed twice with cold PBS, and resuspended in PBS at a density of 1 x 10^10^ CFU/mL. Alternatively following the 3.5-hour outgrowth, *A*. *baumannii* strains were chemically killed by acetone: ethanol as described above prior to washing and resuspending in PBS. Strains were mixed in a 1:1 ratio and sodium dodecyl sulfate, human b-defensin 3, and LL-37 were added at the indicated concentrations, and bacteria were enumerated at the indicated time points by serial dilution and plating. Minimum inhibitory concentrations of kanamycin and gentamicin were determined by spreading 150 μL of an overnight culture of the indicated strain on an LB agar plate, allowing the plate to dry, and placing an MIC test strip (Liofilchem s.r.l.) on the agar surface and incubating the plate at 37° C for 16 hours. MICs were determined by the intersection of the zone of growth inhibition with the test strip.

### Neutrophil depletion

Neutrophil depletion was achieved by intraperitoneal injection of 250 μg rat IgG2b anti-Gr-1 mAb RB6-8C5 (anti-neutrophil antibody) in 100μl PBS 24 hours prior to infection and at the time of infection. Control mice were treated in the same way with an isotype control antibody. The bacterial inoculation was reduced to 3 x 10^7^ CFU to reduce mortality associated with infection of neutrophil depleted mice. The infections were carried out as above using WT bacteria and killed Tn5A7.

### Macrophage depletion

Liposomal encapsulation of clodronate and control (dichloromethylene diphosphonate) in liposomes was performed as described previously [61]. Liposomes were resuspended in 5 ml PBS and stored at 4°C for no more than 24 hours before use. Mice were anesthetized with isoflurane and treated intranasally with 50 μl of clodronate or control liposomes 48 hours prior to infection. Infections were then carried out as described above using WT bacteria and killed Tn5A7.

### Bone marrow-derived macrophage (BMDM) isolation and infection

BMDMs were isolated as previously described [62]. Briefly, bone marrow was flushed from the femurs of C57BL/6 mice, washed, red blood cells were lysed, and the bone marrow suspension was passed through a cell strainer prior to counting. The bone marrow cell suspension was plated at a density of 3 x 10^6^ cells/ml in RPMI medium supplemented with 10% fetal bovine serum (FBS) and 20% LC medium and incubated at 37°C with 5% CO_2_. On day three, 5 ml of RPMI medium supplemented with 10% FBS and 20% LC medium was added to each plate. After six days, 10mL of the media was collected from each plate, pelleted, and replaced with 10 ml of fresh RPMI containing 10% FBS and 20% LC medium. On day seven, cells were collected and plated overnight for the infection assay. BMDMs were infected with the indicated strains at a multiplicity of infection of 15 and infected cells were incubated for four hours. Following incubation, cell culture supernatants were removed and cytokines were quantified as described below.

### Generation of transposon mutants

Transposon mutants were generated using the EZTn5 transposome kit as previously described [24] and plated on LBA supplemented with kanamycin. Eight of the resulting colonies were picked and saved for future analyses.

### Construction of Tn5A7Δ*lpsB* and Tn5A7Δ*pilQ*

Strains Tn5A7Δ*lpsB* and Tn5A7Δ*pilQ* were constructed by recombineering using the protocol described by Tucker, et al [63]. Briefly, primers were designed that include approximately 125 bp of sequence upstream of the gene to be deleted (*lpsB*, A1S_0430 and *pilQ*, A1S_3191) fused to the *tetA* forward primer, CGTCAGGCTGTCCATGCC, and 125 bp of sequence downstream of the gene to be deleted fused to the *tetA* reverse primer, GCCATGACTAAATCACAATGAAG (Integrated DNA Technologies). These primer pairs were used to amplify *tetA* from *A*. *baumannii* AYE genomic DNA. After purification, approximately 10 μg of each amplification product was electroporated into electrocompetent Tn5A7 containing the recombinase plasmid, pAT02. Transformants were recovered at 37°C with shaking in LB containing 2 mM IPTG for 4 hours to induce recombinase expression prior to plating onto LB agar containing tetracycline. Tetracycline-resistant colonies were purified by serial passage and confirmed to have the appropriate deletion by failure to amplify the gene in question and by PCR amplification using a primer that binds within *tetA* and a primer that binds outside of the flanking sequence included in the recombineering primer. The Tn5A7Δ*lpsB* deletion included deletion of the Tn5 disrupting *lpsB* in Tn5A7, thereby rendering this strain susceptible to kanamycin. Plasmid pMU368 was introduced into Ab17978 and Tn5A7Δ*lpsB* by electroporation to confer plasmid-borne kanamycin resistance.

### Complementation of *lpsB* in Tn5A7

The *E*. *coli*-*A*. *baumannii* GFP expression vector pMU125 was modified by digesting with EcoRV to excise GFP and self-ligated to produce pAb001. A 1500 bp fragment including *lpsB* and 500 bp of flanking sequence was amplified from WT genomic DNA using forward primer GCCCGAATTCGCTTCGTATCGCACCAACTC and reverse primer CCCGGATATCTCAATTCAATACACTTTGATATAGCTC. The product was digested with EcoRI and EcoRV and cloned into the cognate sites of pAb001 to produce p*lpsB*. The complementation vector p*lpsB* was introduced into Tn5A7 by electroporation as previously described (24).

### Cytokine measurement

Determination of lung cytokine levels was performed on homogenized whole lungs from infected animals harvested four- and 12- hours post-infection and homogenized in PBS. Samples were normalized to total protein prior to analysis. Determination of systemic cytokine levels was performed on serum isolated from infected mice at the indicated time points. Bone marrow-derived macrophage cytokines were quantified from cell culture supernatant at four hours post-infection. All cytokines were quantified using the MAP Mouse Cytokine/Chemokine Magnetic Bead Panel (Millipore) on a Luminex Flexmap 3D platform (Luminex) according to the manufacturer’s recommended protocol.

### NFκB activation

NF-κB activation in response to *A*. *baumannii* was assessed by Western blotting using anti-mouse NF-κB p65 antibodies (Abcam). RAW264.7 cells were infected with Ab17978/pMU368 propagated in LB or LB supplemented with kanamycin at a multiplicity of infection of 100 for 2 hours. Cells were collected and fractionated following the REAP method described by Suzuki, et al. [64]. The nuclear and cytosolic fractions were separated, normalized to total protein by bicinchoninic acid assay, run on an SDS-PAGE gel, and transferred to nitrocellulose membranes. Membranes were incubated with anti-histone 3 antibody (Cell Signaling) as a nuclear marker, anti- β-actin antibody (Abcam) as a loading control, as well as anti-NF-κB p65 antibody. Blots were imaged with an Odyssey Imaging System (LI-COR Biotechnology). Total NF-κB in the nuclear and cytosolic fraction were quantified by densitometry using ImageJ.

### Histology

Paraffin-embedded mouse tissue sections were stained with hematoxylin and eosin by the Vanderbilt University Medical Center Translational Pathology Shared Resource. Specimens were examined and imaged by K. L. B. who was blinded to the treatment. Images are representative of at least 3 independent experiments.

### Complete blood count

Complete blood counts with five-part differential were performed on EDTA-treated whole mouse blood by the Vanderbilt University Medical Center Translational Pathology Shared Resource.

### Immune cell recruitment

Flow cytometric analyses were performed with total erythrocyte-free lung cells isolated at the indicated times post-infection from individual mice infected with Ab17978 or co-infected as described. Lungs were minced, digested with collagenase and DNase for 30 minutes, and passed through a 70 μm cell strainer prior to erythrocyte lysis. Cells were stained with a myeloid panel that included antibodies against CD45 (clone 104, FITC, eBioscience), CD103 (clone 2E7, PerCP-Cy5.5, BioLegend), CD64 (clone X54-5/7.1, PE, BioLegend), CD11c (clone HL-3, PE-Cy7, BD Pharmingen), Siglec F (clone E50-2440, Horizon PE-CF594, BD Pharmingen), CD11b (clone M1/70, eFluor-450, eBioscience), MHCII (clone M5/114.15.2, BV605, BD Pharmingen), CD24 (clone M1/69, APC, eBioscience), Ly6C (clone AL-21, APC-Cy7, BD Pharmingen), and Ly6G (clone 1A8, Alexa Fluor 700, BD Pharmingen). Analyses were carried out on the 4-laser BD LSR Fortessa (BD Biosciences) at the Vanderbilt Flow Cytometry Shared Resource, and analyses were performed using FlowJo software (Treestar Inc). Myeloid populations were gated according to the strategy of Yu and colleagues [65] and representative gating strategy is shown in S4 Fig.

### Quantification and statistical analysis

Statistical analyses were performed using GraphPad Prism version 6. Mean comparisons were performed using unpaired Welch’s *t*-test or one-way ANOVA adjusted for multiple comparisons. Median survival times were compared using a Kaplan-Meier analysis and log-rank test. P values less than 0.05 were considered statistically significant. Statistical details of experiments can be found in the figure legends.

### Ethics statement

All animal experiments were approved by the Vanderbilt University Institutional Animal Care and Use Committee protocol number M1700072-00 in accordance with the United States Animal Welfare Act and the United States Public Health Service Policy.

Additional methodological details are available in the Supporting Information.

## Supporting information

S1 Table Bacterial strains included in this study. *, references *A*. *baumannii* ATCC 17978 reference genome annotations; AG, aminoglycoside; MIC, minimum inhibitory concentration; Gm, gentamicin; NA, not applicable; ND, not determined. All MIC values are in μg/mL.
(DOCX)

S1 Fig Tn5A7 does not protect mice during systemic infection. Mice were infected with WT mixed with an equal volume of PBS or WT mixed with an equal inoculum of Tn5A7 retro-orbitally and survival was monitored during the 30-hour infection. Sample size was five mice per group. Median survival times were compared using Kaplan-Meier analysis and log-rank test and P<0.05.
(TIF)

S2 Fig Ab17978 growth in kanamycin. WT *A*. *baumannii* was grown in lysogeny broth supplemented with the indicated concentration of kanamycin and growth was assayed over time through measurement of the optical density at 600 nm.
(TIF)

S3 Fig Lung and blood levels of TNFα, IL-6, and IFNγ. The indicated cytokines were quantified from whole lung homogenates and blood of mice at four- and 12-hours following infection with WT propagated in lysogeny broth, Tn5A7 propagated in lysogeny broth containing kanamycin, or co-infected with an equal mixture of the two strains. Columns depict the mean and error bars show standard deviation of the mean. Means were compared with the mean of WT at that time using a one-way ANOVA adjusted for multiple comparisons and no statistically significant differences were observed.
(TIF)

S4 Fig Flow cytometric analysis of mouse lung. Contour plots of windows and gating strategy used for the identification of immune cell populations from a representative infected mouse lung are shown. Gates containing multiple cell populations are numbered (P1-P6). Gates including a single population are labeled with the included cell type.
(TIF)

S5 Fig Eosinophil, NK cell, and lymphocyte recruitment to the lung. Eosinophils, NK cells, and lymphocytes were quantified from the lungs of mice using flow cytometry at four- and 12-hours following infection with WT *A*. *baumannii* propagated in lysogeny broth, Tn5A7 propagated in lysogeny broth containing kanamycin, or co-infected with an equal mixture of the two strains. The y-axis depicts the percentage all CD45-positive cells. Means were compared with the mean of WT at that time using a one-way ANOVA adjusted for multiple comparisons and no statistically significant differences were observed.
(TIF)

S6 Fig Aminoglycoside growth curves. The indicated bacterial strains were grown in lysogeny broth alone or supplemented with 40 μg/mL kanamycin or 50 μg/mL gentamicin and growth was assessed by measuring the optical density at 600 nm over time.
(TIF)
