# Supplementary material for: Broad-spectrum suppression of bacterial pneumonia by aminoglycoside-propagated Acinetobacter baumannii
Source: PLoS Pathog. 2020 Mar 13;16(3):e1008374. doi: 10.1371/journal.ppat.1008374 (PMC7094866; doi:10.1371/journal.ppat.1008374)
Supplement: S1 Table — *, references A. baumannii ATCC 17978 reference genome annotations; AG, aminoglycoside; MIC, minimum inhibitory concentration; Gm, gentamicin; NA, not applicable; ND, not determined. All MIC values are in μg/mL. (DOCX) [file ppat.1008374.s001.docx]

| **Strain** | **Description** | **Annotation^*^** | **Source** | **Mechanism of AG resistance** | **Km MIC** | **Gm**  **MIC** | **Reference** |
| --- | --- | --- | --- | --- | --- | --- | --- |
| AB 17978 | *A. baumannii* ATCC 17978 used as wild type, originally isolated from fatal meningitis |  | ATCC | NA | 0.75 | 0.38 | [1] |
| Tn5A7 | AB 17978 withTn5 inserted into Putative glycosyltransferase (*lpsB*) | A1S_0430 | Previously generated | AG 3'-phospho-transferase | 128 | 1.5 | [2] |
| Tn20A11 | AB 17978 with Tn5 intergenic insertion between a major facilitator superfamily transporter and *esvB* | A1S_1231, A1S_1232 |  | AG 3'-phospho-transferase | 128 | 3.0 | This study |
| AB *ΔlpsB* | AB 17978 *lpsB::km* | A1S_0430 |  | AG 3'-phospho-transferase | >256 | 3 | [2] |
| Tn5A7 *ΔpilQ/*pAT02 | Tn5A7 *pilQ*::tetM | A1S_3191 |  | AG 3'-phospho-transferase | 64 | 2 | This study |
| Tn5A7 p*lpsB* | Tn5A7 containing *lpsB* complementation vector |  |  | AG 3'-phospho-transferase | ND | ND | [2] |
| Tn5A7 *ΔlpsB* | Tn5A7 *lpsB::km* | A1S_0430 |  | NA | ND | ND | This study |
| Tn5A7 *ΔlpsB*/pMU368 | Tn5A7 *ΔlpsB* containing pMU368 |  |  | AG 3'-phospho-transferase | ND | ND | This study |
| AB 17978/pMU368 | AB 17978 containing pMU368 |  |  | AG 3'-phospho-transferase | 96 | 0.38 | This study |
| Tn1 | AB 17978 with Tn5 intergenic insertion between a high-affinity phosphate transport protein (*pstB*) and putative periplasmic protease | A1S_2444,  A1S_2445 |  | AG 3'-phospho-transferase | 96 | 0.75 | This study |
| Tn2 | AB 17978 with Tn5 inserted into a putative acetyl-CoA acetyltransferase | A1S_2842 |  | AG 3'-phospho-transferase | >256 | 3 | This study |
| Tn3 | AB 17978 with Tn5 inserted into a hypothetical protein | A1S_2982 |  | AG 3'-phospho-transferase | >256 | 0.75 | This study |
| Tn4 | AB 17978 with Tn5 inserted into a hypothetical protein | A1S_2250 |  | AG 3'-phospho-transferase | 64 | 0.5 | This study |
| Tn7 | AB 17978 with Tn5 inserted into a putative malate dehydrogenase | A1S_3025 |  | AG 3'-phospho-transferase | >256 | 2.0 | This study |
| Tn8 | AB 17978 with Tn5 inserted between a putative lipoprotein-34 precursor (NlpB) and a phosphoribosylaminoimidazole-succinocarboxamide | A1S_3424,  A1S_3425 |  | AG 3'-phospho-transferase | >256 | 1.0 | This study |
| AB 301 | *A. baumannii* 301-0294 used as an alternative wild type, originally isolated from bloodstream infection |  | Dr. Anthony Campagnari (Buffalo, NY) | NA | ND | ND | [3] |
| *S. aureus* JE2 | Methicillin-resistant USA300 clone |  | Dr. Paul Fey (Omaha, NE) | NA | ND | ND | [4] |
| *P. aeruginosa* PA01 | Wound isolate |  | Dr. Andrea Battistoni (Rome, Italy) | NA | ND | ND | [5] |
| *K. pneumonia* ATCC 43816 | Serotype 2 strain |  | Dr. R. Stokes Peebles (Nashville, TN) | NA | ND | ND | [6] |
| AB17978*Δhcp* | Ab 17978 with  *hcp* replaced by gentamicin-resistance cassette |  | Dr. M. Feldman | Gm 3-N-acetyl-transferase | 3.0 | >256 | [7] |
| Ab17978/ p.*luxABCDE*.MU368.tet | Ab 17978 with p.*luxABCDE*.MU368.tet |  |  | AG 3'-phospho-transferase | 64 | 0.125 | [8] |

**References**

1. Baumann P, Doudoroff M, Stanier RY. A study of the Moraxella group. II. Oxidative-negative species (genus Acinetobacter). J Bacteriol. 1968;95(5):1520-41.

2. Hood MI, Becker KW, Roux CM, Dunman PM, Skaar EP. genetic determinants of intrinsic colistin tolerance in *Acinetobacter baumannii*. Infect Immun. 2013;81(2):542-51.

3. Russo TA, Beanan JM, Olson R, MacDonald U, Luke NR, Gill SR, et al. Rat pneumonia and soft-tissue infection models for the study of *Acinetobacter baumannii* biology. Infect Immun. 2008;76(8):3577-86.

4. Fey PD, Endres JL, Yajjala VK, Widhelm TJ, Boissy RJ, Bose JL, et al. A genetic resource for rapid and comprehensive phenotype screening of nonessential *Staphylococcus aureus* genes. mBio. 2013;4(1):e00537-12.

5. Karkhoff-Schweizer RR, Schweizer HP. Utilization of a mini-Dlac transposable element to create an alpha-complementation and regulated expression system for cloning in *Pseudomonas aeruginosa*. Gene. 1994;140(1):7-15.

6. Bakker-Woudenberg IA, van den Berg JC, Vree TB, Baars AM, Michel MF. Relevance of serum protein binding of cefoxitin and cefazolin to their activities against *Klebsiella pneumoniae* pneumonia in rats. Antimicrob Agents Chemother. 1985;28(5):654-9.

7. Weber BS, Miyata ST, Iwashkiw JA, Mortensen BL, Skaar EP, Pukatzki S, et al. Genomic and functional analysis of the type VI secretion system in Acinetobacter. PLoS One. 2013;8(1):e55142.

8. Juttukonda LJ, Chazin WJ, Skaar EP. *Acinetobacter baumannii* Coordinates Urea Metabolism with Metal Import To Resist Host-Mediated Metal Limitation. mBio. 2016;7(5).
