# Supplementary figures and images for: Broad-spectrum suppression of bacterial pneumonia by aminoglycoside-propagated Acinetobacter baumannii
Source: PLoS Pathog. 2020 Mar 13;16(3):e1008374. doi: 10.1371/journal.ppat.1008374 (PMC7094866; doi:10.1371/journal.ppat.1008374)

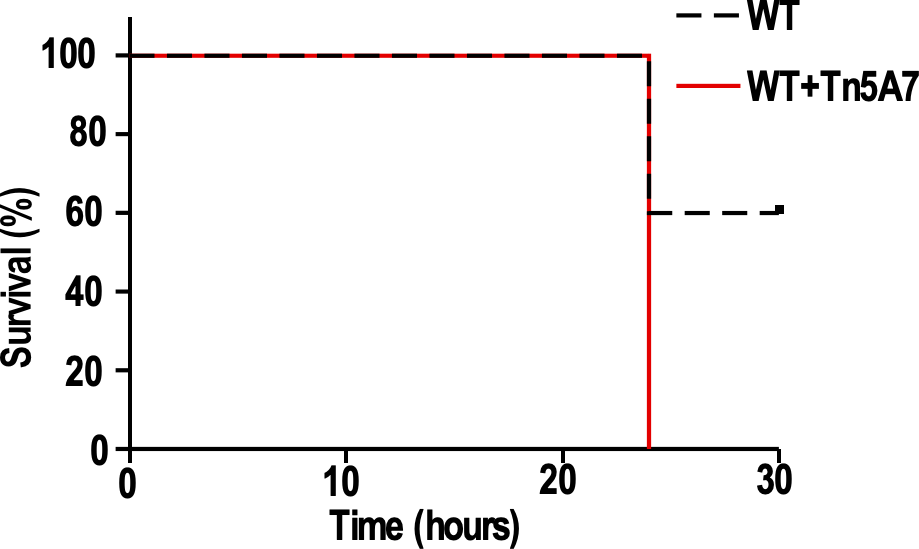

Supplement: S1 Fig — Mice were infected with WT mixed with an equal volume of PBS or WT mixed with an equal inoculum of Tn5A7 retro-orbitally and survival was monitored during the 30-hour infection. Sample size was five mice per group. Median survival times were compared using Kaplan-Meier analysis and log-rank test and P<0.05. (TIF) [file ppat.1008374.s002.tif]

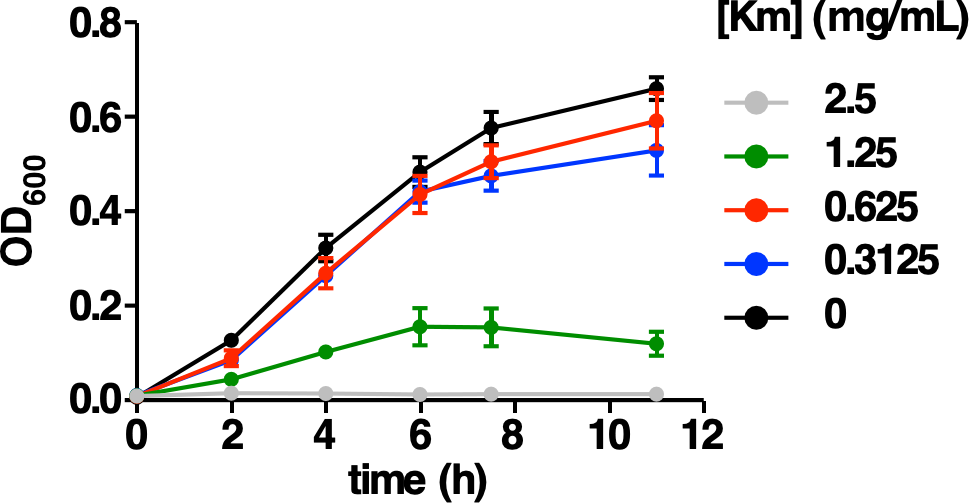

Supplement: S2 Fig — WT A. baumannii was grown in lysogeny broth supplemented with the indicated concentration of kanamycin and growth was assayed over time through measurement of the optical density at 600 nm. (TIF) [file ppat.1008374.s003.tif]

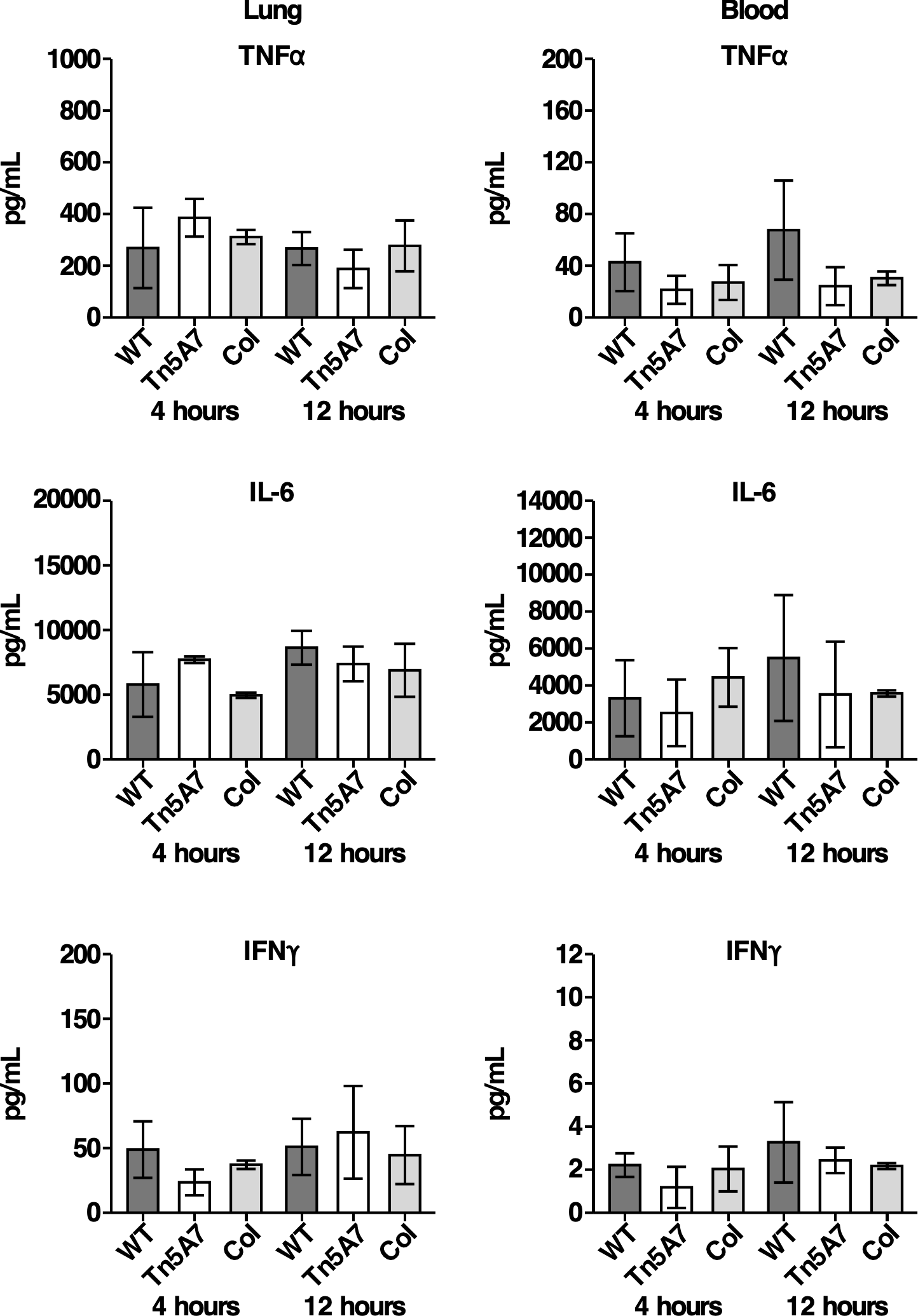

Supplement: S3 Fig — The indicated cytokines were quantified from whole lung homogenates and blood of mice at four- and 12-hours following infection with WT propagated in lysogeny broth, Tn5A7 propagated in lysogeny broth containing kanamycin, or co-infected with an equal mixture of the two strains. Columns depict the mean and error bars show standard deviation of the mean. Means were compared with the mean of WT at that time using a one-way ANOVA adjusted for multiple comparisons and no statistically significant differences were observed. (TIF) [file ppat.1008374.s004.tif]

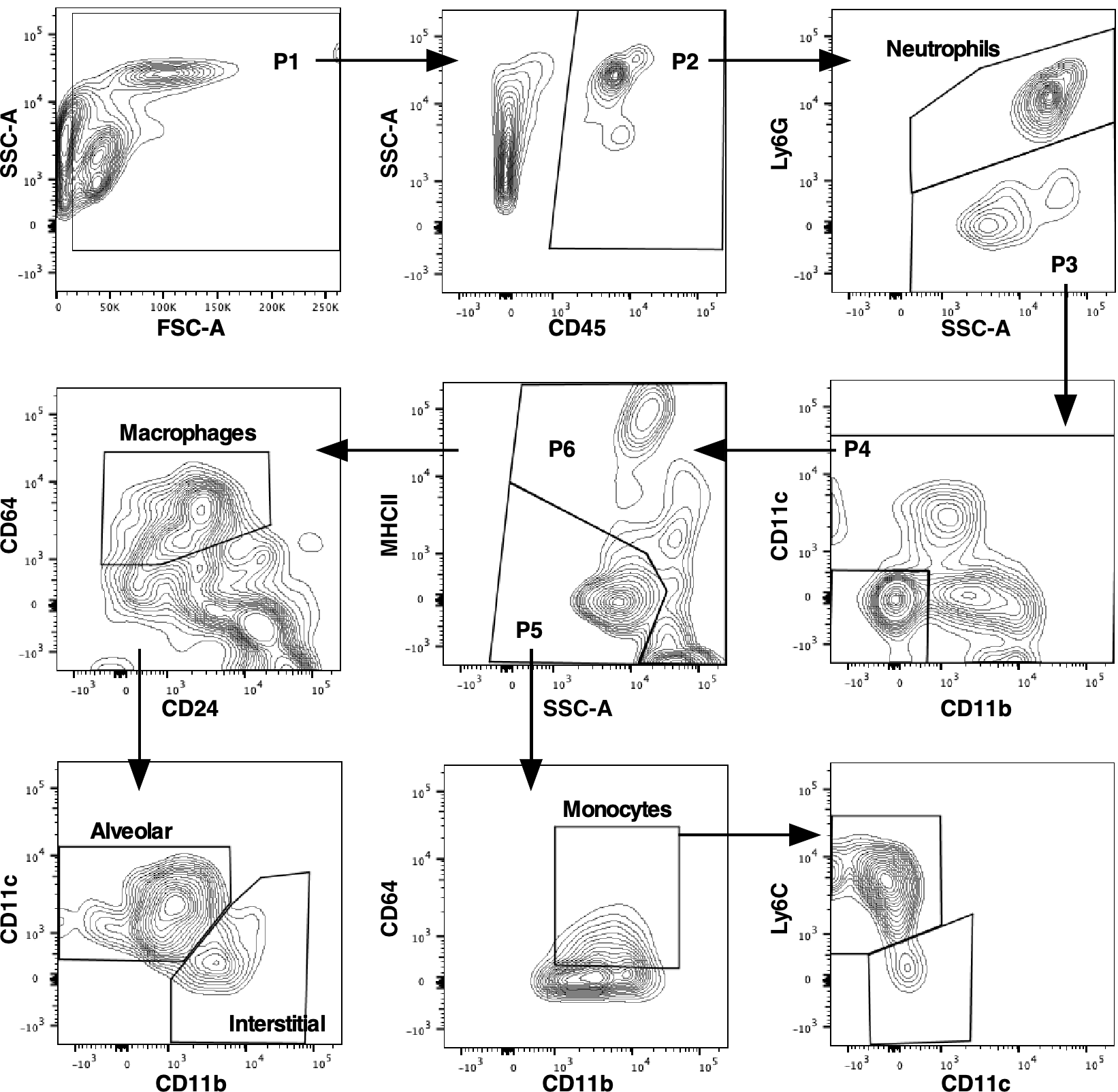

Supplement: S4 Fig — Contour plots of windows and gating strategy used for the identification of immune cell populations from a representative infected mouse lung are shown. Gates containing multiple cell populations are numbered (P1-P6). Gates including a single population are labeled with the included cell type. (TIF) [file ppat.1008374.s005.tif]

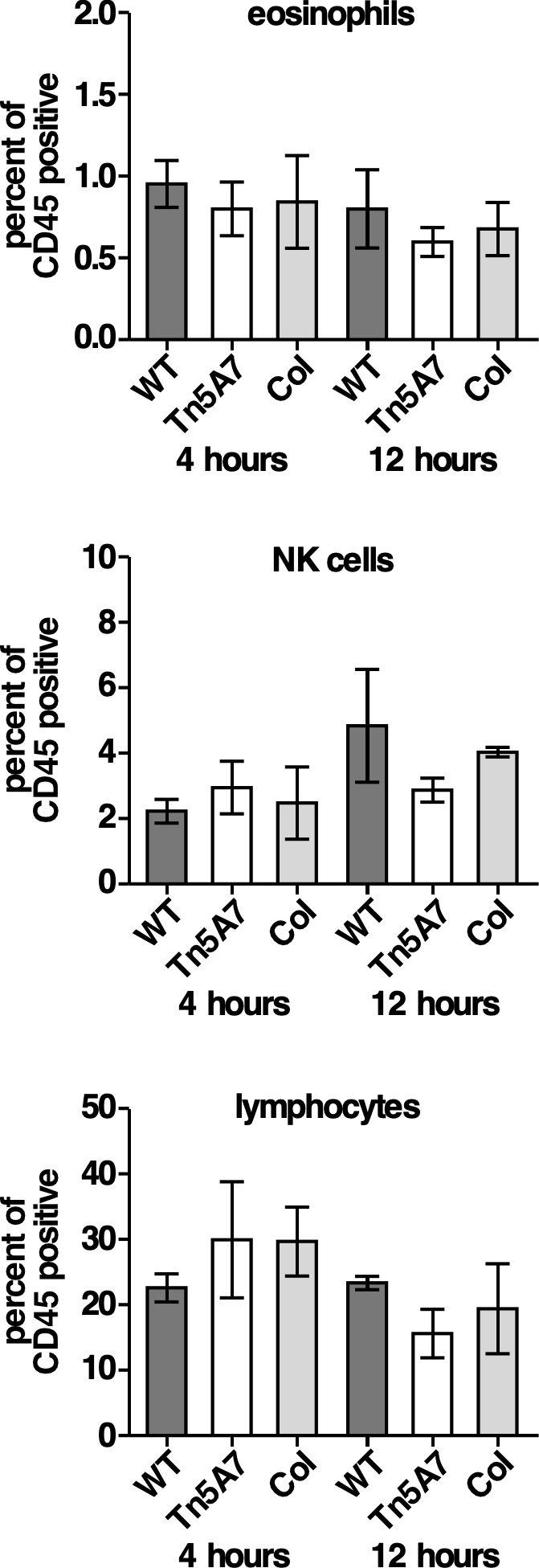

Supplement: S5 Fig — Eosinophils, NK cells, and lymphocytes were quantified from the lungs of mice using flow cytometry at four- and 12-hours following infection with WT A. baumannii propagated in lysogeny broth, Tn5A7 propagated in lysogeny broth containing kanamycin, or co-infected with an equal mixture of the two strains. The y-axis depicts the percentage all CD45-positive cells. Means were compared with the mean of WT at that time using a one-way ANOVA adjusted for multiple comparisons and no statistically significant differences were observed. (TIF) [file ppat.1008374.s006.tif]

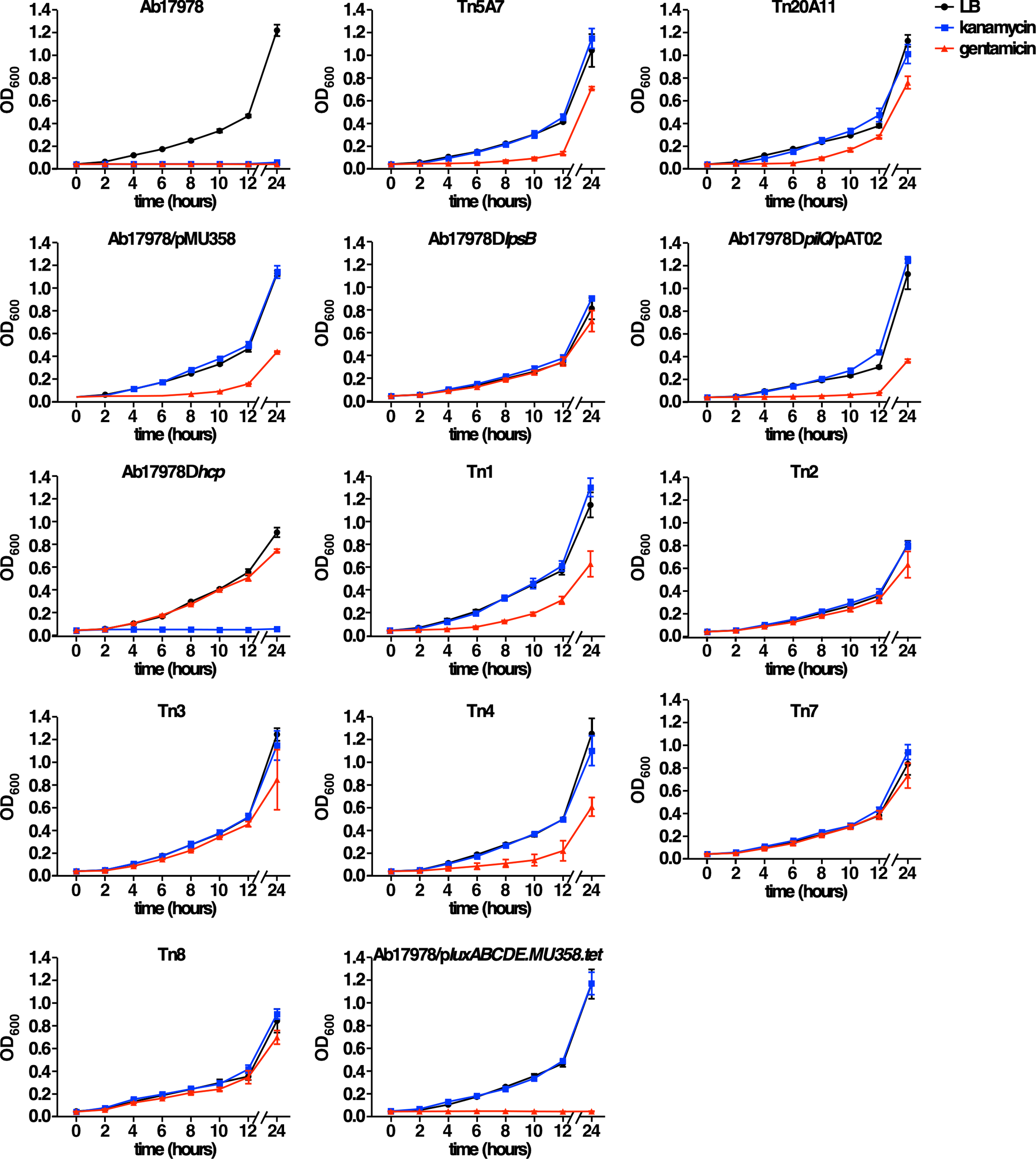

Supplement: S6 Fig — The indicated bacterial strains were grown in lysogeny broth alone or supplemented with 40 μg/mL kanamycin or 50 μg/mL gentamicin and growth was assessed by measuring the optical density at 600 nm over time. (TIF) [file ppat.1008374.s007.tif]
